# Supplementary material for: Phantom rivers filter birds and bats by acoustic niche
Source: Nat Commun. 2021 May 24;12:3029. doi: 10.1038/s41467-021-22390-y (PMC8144611; doi:10.1038/s41467-021-22390-y)
Supplement: Supplementary file 1 — Supplementary Information [file 41467_2021_22390_MOESM1_ESM.pdf]

Supplementary Information for

Phantom rivers filter birds and bats by acoustic niche

D. G. E. Gomes, C. A. Toth, H. J. Cole, C. D. Francis, J. R. Barber

Correspondence to:

dylangomes@u.boisestate.edu

jessebarber@boisestate.edu

This PDF file includes:

Materials and Methods

Figs. S1 to S14

Tables S1 to S13

References

Materials and Methods ... 3
- Experimental setup ... 3
- Environmental parameter quantification ... 6
- Bird abundance ... 8
- Bird foraging trials ... 14
- Bat activity ... 15
- Bat foraging trials ... 19
- A note on treatment vs continuous analyses ... 20

Fig S1. ... 25
Fig S2. ... 26
Fig S3. ... 27
Fig S4. ... 28
Fig S5. ... 29
Fig S6. ... 30
Fig S7. ... 31
Fig S8. ... 32
Fig S9. ... 33
Fig S10. ... 33
Fig S11. ... 34
Fig S12. ... 35
Fig S13. ... 36
Fig S14. ... 37
Table S1. ... 39
Table S2. ... 41
Table S3. ... 51
Table S4. ... 53
Table S5. ... 54
Table S6. ... 56
Table S7. ... 60
Table S8. ... 61
Table S9. ... 62
Table S10. ... 64
Table S11 ... 65
Table S12 ... 67
Table S13 ... 68
References ... 69

Materials and Methods

IACUC approval: All work described below was approved by the Boise State Institutional Animal Care and Use Committee: AC15-021

Experimental setup

Noise playback setup:

We suspended speakers from tripods made of 4.5 m metal conduit to elevate them 3-3.5 m from the ground to improve noise propagation (**Fig S2**). River noise was broadcast at each site via two Octasound SP820A speakers (35 Hz to 20 kHz ± 10 dB; KDM Electronics Incorporated, Ajax, ON, Canada), whereas shifted noise was broadcast at each site via three Octasound SP800A speakers (40 Hz to 20 kHz ± 12 dB; KDM Electronics Incorporated). This discrepancy was designed to account for differences in propagation due to attenuation of the different spectra of our playbacks. That is, we estimated a similar amount of land area would be exposed to the noise treatments (**Fig S3**). To account for differences in infrastructure between treatments, river noise sites had an additional dummy tower (**Fig S3; S4**) and dummy solar panel placed in the same location as found on shifted-noise sites. Control sites consisted of three dummy towers each paired with a dummy solar panel, placed according to the same spacing as treatment sites. We constructed dummy speaker towers by hanging large metal speaker horns (0.41 m diameter; Dayton Audio RPH16) from tripods made of 4.5 m PVC piping (**Fig S4**). We used particle board (1 x 1.5 m) spray-painted glossy black to mimic solar panels (see below for use of solar panels).

Each speaker was powered by an amplifier (AD1200.1, PRV audio) connected to two 12V deep cycle RV/marine batteries (DURDC12-100P; Duracell) connected in parallel. Solar panels (Suniva OPT285-60-4-100; MidNite Solar Inc., WA, USA; **Fig S2**) charged the deep cycle batteries via solar controllers (The Kid 30A MPPT; MidNite Solar Inc., WA, USA), which also relayed power from the batteries to the amplifier. Roland R05 players relayed sound files from SD cards to the amplifiers, and were powered for multiple weeks at a time via LiFePO3 (3.3V, Batteryspace, CA, USA) batteries. Amplifier settings were as follows: 'over clip' was set to "off" position, 'H.P.F.' was set to minimum value (10 Hz), 'L.P.F.' was set to maximum value (25 kHz), and all three equalizer settings were set to 0 dB.

Creating acoustic environment playback files:

We recorded natural river noise to produce field playback files in June of 2016. We recorded river noise at three locations along Trail and Hyndman creeks near Sun Valley, ID using a Zoom H4N Pro recorder (Zoom North America Inc., Hauppauge, NY, USA) connected to a Rode NT1A condenser microphone (Rode, Silverwater, NSW, Australia) positioned approximately 5 m from the river's edge. This resulted in three, approximately 1-hour long files saved in an uncompressed WAV format with a 48 kHz sample rate and 16-bit depth. We screened all files for all non-river noise (e.g. insects, birds) in the program Audacity (Audacity Team 2017) and removed these instances using the cross-fade function. We standardized the runtime of all river files to 45 minutes, as this was the length of the shortest playback file following the removal of non-relevant sounds. Finally, each file was normalized to an amplitude of -2 dB.

To create the "shifted" playbacks we applied a high-pass filter to each playback file at 2 kHz in Adobe Audition CC 2017 (Adobe Systems, San Jose, CA, USA), then used the Frequency Band Splitter to apply band-specific amplifications to two bands: 2-14 kHz and 14-24 kHz. Each band received a +4 and +5 dB amplification, respectively, following power calculations below.

Next, we compared the power spectra of each playback file to a 'typical' songbird audiogram to ensure that normal and shifted playback files presented a similar sensory impact on the local bird community. We created an audiogram for birds based on values adapted from Dooling (2002)[1] for songbird species whose ranges overlapped our study sites. We extracted threshold values for American robin (*Turdus migratorius*), brown-headed cowbird (*Molothrus ater*), chipping sparrow (*Spizella passerina*), dark-eyed junco (*Junco hyemalis*), red-winged blackbird (*Agelaius phoeniceus*), song sparrow (*Melospiza melodia*), and western meadowlark (*Sturnella neglecta*) as exemplars. We fit the consolidated values with a quadratic function[1], providing audiograms against which the power spectra of the playback files could be compared.

To compare the power of normal and shifted playbacks with respect to songbird hearing, we played the first two minutes of each playback file in an anechoic room at Boise State University at approximate field amplitudes (i.e. ~97 dBA for shifted, ~95 dBA for phantom at 2 m) using our field playback equipment: a Roland R05 player linked to a AD1200.1-2 amplifier (PRV Audio, Fort Lauderdale, FL, USA) and an Octasound SP820A speaker. We recorded these playbacks as uncompressed WAVs at a 44.1 kHz sample rate and 16 bit-depth using a Rode NT1A condenser microphone positioned 2 m from the speaker, connected to a PC computer running Audacity 2017. We then used custom Matlab code (Daniel Mennitt) to calculate the

power within each third-octave band for each file, and used these values to calculate the difference in power for each band to the area under the curve for the respective songbird audiogram between 160 Hz and 16 kHz. Each shifted file was compared to its phantom counterpart and deemed equal if the total power difference was within 3 dB (i.e. the standard error of the microphone). All shifted playbacks were within 1.38 dB of their phantom counterparts.

We used Adobe Audition CC 2017 to create the full-length playback files to be used in the field. These playback files consisted of each 45-minute recording arranged linearly in a random order, with each individual river recording used twice per playback file (no file occurred back-to-back). The order of shifted recordings matched those of their phantom counterparts. We applied a 5 s crossfade between each subsequent recording and applied a 7 s fade in/out to the full-length file to avoid clipping when looped. These files were once again exported in uncompressed WAV format at a sample rate of 48 kHz and a 16-bit depth. In total, this resulted in four playback files – phantom and shifted river – each of approximately 4:30:00 in duration.

Environmental parameter quantification

Acoustic environment quantification:

The acoustic environment was quantified with long-term recordings from Roland R05 recorders (hereafter referred to as an ARU: acoustic recording unit). ARU settings were as follows: 44.1

kHz sampling rate, 128 kbps MP3 recording mode, input gain was set to 55, mic gain = 'H', limiter = 'off', and low cut = 'off.'

We used different timeframes to quantify the local acoustic environment for analyses of bird abundance and bat activity, reflecting taxa-specific differences in behavior and space use. Given that birds establish breeding territories that they inhabit almost exclusively throughout the breeding season (and thus, territorial birds on our site would be experiencing a full day's variance in the acoustic environment's frequency and amplitude values), we intended to use integrated 24-hour measurements of both SPL and spectrum in our models of bird abundance. However, dawn chorus will be full of singing birds - increasing both the amplitude and frequency of background noise. In order to avoid circular findings (i.e., more singing birds equals more birds), we excluded these times of high bird activity (0600-1100). Bats, on the other hand, are likely travelling to/through these sites from their day roosts elsewhere, and likely experience the sites' acoustic environments for only a number of hours each night. Thus, we used nightly averaged SPL and frequency values when modelling bat activity. We quantified 'nightly' hours as those between 2100 and 0600, corresponding roughly to sunrise and sunset times in the region during the course of the study.

Percent riparian vegetation:

The local amount of riparian vegetation may influence both bird abundances[7] and bat activity[8]. Thus, we quantified the proportion of riparian vegetation surrounding sampling locations as a location-specific parameter in our models. We used high spatial resolution land-cover data to manually create polygon vertices surrounding all patches of riparian vegetation (defined as non-

coniferous trees and non-*Artemisia* ground cover) within a 100-meter radius of each sampling location. Following riparian vegetation classification, we calculated the proportion of land-cover within a 100 m radius of each sampling location that was riparian vegetation using 'Tabulate Intersection' in ArcMap 10.2 (Esri, CA, USA).

Moon phase:

The phase of the moon is known to affect the activity of at least some species of bats[9,10]. Thus, we included the phase of the moon (represented by the proportion of the moon visible at midnight), by date, in our models of bat activity. We sourced these data from the U.S. Naval Observatory.

<u>Bird abundance</u>

Issues of detectability in avian point counts:

The detection of birds during point counts can be problematic when hoping to estimate the abundance of birds on the landscape. We employed various techniques that we describe below to correct for both standard detection issues, and detection issues in background noise.

Detectability over distance - distance truncation

We truncated our counts to 50 m, because at this limit it is likely that most birds can be reliably detected[11,12], given that they are singing (see next paragraph). We opted to not use a distance-based model for detectability given the nature of our river sites. Because the habitat is not homogenous, the probability that a bird is detected will not fall off evenly with distance. This situation violates the assumptions of distance-based models. Distance truncation also ensured that birds counted in one location did not overlap with birds counted in another (see *'Data collection locations'* above for distances between locations).

Observer detectability in noise

Counting birds in noisy places raises a critical problem that can be difficult to overcome. Here, we turned speakers off during counts. This largely eliminates high sound level environments that are disruptive to detecting birds (**Fig. S6**)[13]. Additionally, we used three different methods to triangulate on whether or not the patterns we have measured are real, or some artefact of detection probabilities. Firstly, we included background noise levels during point counts as a covariate in a removal model. This allowed the data to tell us how varying sound levels affected our ability to detect birds. Secondly, we created a point-count experiment with our observers to directly calculate these same detection probabilities under various noise conditions. Thirdly, all of our 'regular' point count efforts in the field were paired with an equal number of 'unimodal', vision-only point counts. That is, we significantly limited our ability to detect birds auditorily, and focused on visual detections. All three of these methods consistently converge on the same results (see **Table S1**).

Detectability over time - removal models

An additional issue when counting birds is that the detection probability changes with species and/or location due to the probability that a bird will sing[14]. It is well-known that the probability that a bird will sing can vary seasonally and daily. Thus, we used various combinations of ordinal date, time after sunrise, and scaled quadratic versions of those as predictors to explain the singing rate (see **Table S11**). Additionally, the probability of detecting a singing bird can change with the acoustic environment. Loud rivers can partially mask singing birds, and make them less detectable. Thus, we included background sound pressure level (3-min Leq dBA; hereafter 'LEQ') as an additional covariate. Then we used AIC to select the best fitting model, which included the quadratic time after sunrise term and the LEQ term[15]. Thus, this detection probability `p`, was modelled with a conventional removal model, and `p` was used as an offset in our negative binomial (NB) generalized linear mixed effects models[14–16].

Point count experiment

The ability of human observers to detect birds during loud point counts is a known issue[11], yet some sound pressure levels are acceptable for point counting[13]. In our experiment we turned off speakers at our noise treatment sites for point counts. This alleviated differences in background sound levels (and thus detectability) between control and treatment sites (**Fig S6)**.

Because playback speakers were turned off during counts, most of our point counts were conducted in relatively quiet conditions (mean = 48.7 dBA, sd = 9.2 dBA, median = 47 dBA; **Fig S6**). However, some point-count locations (such as positive control sites) were naturally loud (up to 78.8 dBA). Thus, as one approach to control for this problem, we estimated the ability of our

point count observer to correctly identify birdsong in such locations by performing a controlled detectability experiment under varying background sound levels.

We created six playback files consisting of songs from seven of the most-common birds detected during point counts in the 2017 season, sourced from the Macaulay Library: American robin (*Turdus migratorius*; ML 203260), house wren (*Troglodytes aedon*; ML 144011), lazuli bunting (*Passerina amoena*; ML 49753), northern flicker (*Colaptes auratus*; ML 63068), song sparrow (*Melospiza melodia*; ML 144005), spotted towhee (*Pipilo maculatus*; ML 177208), and warbling vireo (*Vireo gilvus*; ML 110999). Each birdsong playback was six minutes in length (to mimic two, three-minute point counts), with 21 songs/speaker, three songs per species/speaker, for a total of 42 songs across two speakers. Each instance of birdsong was separated by at least one second of silence, and all were normalized to 0.1 dB.

For this experiment, point count observers were situated in an open landscape at a time of year when birdsong had largely ceased (e.g. following the breeding season) to minimize the occurrence of natural birdsong during the experiment. In 2017, two point counters performed the experiment in sagebrush steppe in autumn, and in 2018 four point counters performed the experiment in an alfalfa field in late July. Two FoxPro speakers (Wildfire; Lewistown, PA, USA) were placed on each side of the point counter – one at 25 m from the point counter and the other at 50 m (i.e. within our truncation distance) – that broadcast birdsong during each experimental trial. We calibrated the volume of each bird playback speaker such that birdsong was broadcast at biologically relevant amplitudes of 90 dBA at 1 m. An Octasound SP820A was suspended approximately 2-3 m from the point counters to broadcast river noise (using the same

playback file broadcast in the primary study). Point counters performed birdsong identification under several acoustic conditions: natural ambient levels (i.e. no river playback), as well as river noise broadcast at 47 dBA, 51 dBA, 63 dBA, and 73 dBA - the highest sound pressure level (1 hr LEQ) recorded at positive control sites in 2017. We calibrated river playback amplitudes and ambient condition sound pressure levels as a 30 second Leq (dBA) using a Larson Davis (model 824; Depew, NY, USA).

Each point count observer performed bird identification for a randomly selected (without replacement) combination of birdsong playlist and river-playback amplitude. The order in which these point counts were performed was also randomly determined. Misidentified or unheard instances of birdsong by the point counter were recorded as a 0, while correct identifications were marked as a 1. To model how river noise affected birdsong identification, we used the software JMP v14.2 (SAS Institute) to perform a logistic regression on the pooled results of all point counters, with sound level as the predictor. This generated a probability formula for the correct identification of birdsong with the following equation:

$$\text{Detection probability} = \left(1 + e^{-(a-bx)}\right)^{-1}$$

where a = 11.84; b = 0.154; x = 3-min Leq (dBA)

During each point count we recorded sound pressure levels (dBA) as a 3-min Leq (MicW; i436; Beijing, China), which was then used in the equation above to calculate detection probabilities in the various acoustic environments (see **Fig S7**). We used this probability as an offset in generalized linear mixed effects models with raw point count data.

Earplugs & earmuffs

Observers wore earplugs and over-the-ear earmuffs for approximately half of the point counts to limit the observer to visual-only observations in an acoustically-challenging environment (**Table S12)**. This served as a comparison of detectability, since birdsong is more difficult to hear in some sites but not others, whereas vision-only detectability should not vary with sound level.

We ran the same bird abundance models with vision-only counts to exclude the possibility of auditory detections being disrupted by varying levels of noise as an additional way to validate our findings. When this vision-only model is run, daily sound pressure level (SPL; dBA L50) and spectral overlap between background frequency and birdsong are again significant predictors, further validating the directions of these patterns in our global model (**Table S1**).

Species-level analyses

We created similar abundance models (as the global model presented in the main text) for 26 individual species of birds. Dusky flycatchers and song sparrows both avoid high sound levels. Yet, Brewer's sparrows were more abundant in high intensity environments (**Table S2**), even though the same species avoided energy-extraction noise in another experimental study[3]. This result suggests that Brewer's sparrows might use river noise as a source of information to predict the presence of habitat shielded from predators, as birds that stay in noisy areas can experience reduced nest predation[17]. It is also possible that this sagebrush-specialist species benefits from

adjacent riparian areas, which can be predicted by the sounds of running water. Indeed, other taxa show attraction to such habitat selection cues[18].

In our model that included all species, birds did not respond to the median frequency of the acoustic environment, yet nine individual species did – with seven of these species responding negatively to increasing background frequency. These patterns may be a result of deficits to sound localization behavior as small-headed songbirds rely upon higher frequencies for inter-aural level cues (4 kHz and above)[19].

When testing the effects of masking on birdsong, lazuli buntings definitively avoided background noise that spectrally overlaps their song, and other species trended in that direction as well **(Table S2)**. Counter-intuitively, orange-crowned warblers and spotted towhees were more abundant with greater spectral overlap – as if they were attracted to background spectra that were similar to their song. It is possible that these birds use frequencies similar to their own vocalizations (e.g. conspecifics) as indirect information about habitat quality. In this case, river noise could be acting as a misleading cue[20] by attracting birds to habitat that is not higher quality. Alternatively, these animals may be seeking acoustic refuge from eavesdropping predators, as has been suggested for frogs that call near waterfalls to avoid bat predation[21].

Bird foraging trials

Caterpillar scoring

We checked caterpillars on the second, fourth, and fifth days (the first day being the placement day and the fifth day being the last day) for signs of predation. Thus, we totaled 3600 caterpillar-days during the experiment. When signs of predation were present, caterpillars were removed from the substrate and all remaining caterpillars were removed from each site and scored on the fifth day. The type of predator was assessed separately by 2-3 individuals who were blind as to which site the caterpillars came from. If the assessments did not match across observers, predation values were averaged[22]. That is, if two of the three observers scored the caterpillar as being predated by birds, then that caterpillar received a score of 0.67 instead of 1. Each observer scored whether the caterpillars were not predated (NP), predated by birds (B), predated by arthropods (A), or predated by mammals (M), which were all rodents. Here we focus on avian predation (**Fig S8**), since the focus of this paper is on bird and bat predators, not arthropods or other non-bat mammals.

Bat activity

Each SM3 was programmed to automatically record bat activity for five and a half hours each night. However, the timing of these recordings differed between the 2017 and 2018 seasons. Triggered recording began 30 minutes before sunset in both years, but extended for two hours following sunset in 2017 and three hours following sunset in 2018. Triggered recording resumed three hours before dawn in both years, continuing until sunrise in 2017. However, a one-hour long recording break was programmed to occur two hours before sunrise in the 2018 season to

accommodate sonic recording for a separate study (**Fig S10**). Ultrasonic recordings were made at a 256 kHz sample rate.

We used SonoBat v4.3 (SonoBat, CA, USA) to categorize bat calls to the species level using the Western Wyoming classifier set. Acceptable call quality was set to 0.8, a maximum of 32 calls were considered per sequence, and a 10 kHz high pass filter applied. We aggregated call sequence identifications from the "1st" column of all resultant SonoBat output sheets by sample night for each detected species to use as a metric of species-level activity in further analyses, as laboratory tests showed this classification to be the most robust for files with high background noise (see below). We excluded 122 bat nights (66 in 2017, 56 in 2018) from the resulting dataset as the placement of the SM3 coincided with nighttime insect trapping using a UV bucket trap, which have been shown to decrease the activity of light-averse bat species[23].

Bat detection probabilities in noise

We employed two control experiments to ensure that neither the SM3 recording units nor SonoBat were impaired by the acoustic environment playbacks. First, we performed playbacks of echolocation calls from two relatively low-frequency species that are common on our study sites under similar acoustic environment conditions as found on experimental sites. This was to confirm that the detectors were properly triggered under field-playback conditions. Second, we tested the ability of the classification software we used, SonoBat, to correctly classify bats in varying noise conditions.

Passive acoustic monitor 'triggering' in noise

We chose *Antrozous pallidus* and *Eptesicus fuscus* (peak frequency approximately 30 and 28 kHz, respectively) sonar calls to playback in this experiment as our speakers were capable of faithfully reproducing the frequencies of these bats' echolocation cries. We selected two call sequences per species from our datasets – one of high amplitude and one of low amplitude – to ensure that even distant or off-axis bat passes were still able to trigger a recording under high background sound levels.

We performed playbacks in an anechoic room at Boise State University. We broadcast all echolocation call sequences using an UltraSoundGate Player (BL Pro; Avisoft Bioacoustics; Glienicke/Nordbahn, Germany). Both the SM3 microphone and the UltraSoundGate Player were positioned on tripods 1 m from the floor. For *A. pallidus* calls the microphone and speaker were positioned 80 cm apart, while for *E. fuscus* they were positioned 40 cm apart. These spacings were determined by the quality of the recorded call in silence, with the speaker and microphone on-axis with each other.

To simulate the most difficult acoustic conditions under which the SM3 would be required to trigger in the field, we used Adobe Audition CC 2017 to isolate a ~1 s clip of the highest sound level sections found in each of the river and shifted-river playback files and created two "peak" playback files (i.e. peak phantom river and peak shifted river) that consisted only of these sections on loop. We broadcast these files from an Octasound SP820A speaker (suspended approximately 3 m above the ground) using a Roland R05 player and a PRV Audio AD1200.1-2 amplifier at an amplitude of 80 dB(A) as measured at the SM3 microphone using a Larson Davis 824 sound level meter. This amplitude approximates some of the highest sound level playback

conditions under which the SM3 operated in the field, as the highest 1hr LEQ values recorded at SM3 locations on phantom river and shifted river playback sites during the entire study were 77.0 and 82.6 dB(A), respectively.

We played each bat call sequence directly at the SM3 microphone in four directions – facing head on, 90 degrees to the left, 90 degrees to the right, and directly behind. We broadcast each call at as high of an amplitude as was possible without clipping, resulting in the following dB values, as measured at 10 cm from the speaker: *A. pallidus*: 82.1 and 79.1 dB and *E. fuscus*: 78.5 and 73.5 dB. Given that bats of the family Vespertillionidae have been recorded producing echolocation cries of over 122 dB at 10 cm from the bat's mouth[24], our playbacks therefore approximate bats that are either off-axis or much further away (upwards of 28 m) from the microphone. We broadcast these call sequences under three acoustic environment conditions: ambient (i.e. no playback; 33.5 dBA), peak phantom river, and peak shifted river. We manually confirmed that the SM3 triggered and recorded the full echolocation call sequence following playbacks.

SonoBat classification in noise

To investigate if the classification ability of SonoBat was compromised by high background noise we performed a second control experiment wherein we applied shifted-river noise to call sequences recorded under relatively quiet conditions. We isolated a section of shifted-river noise recorded by the ultrasonic channel of the SM3 at our highest sound level SM3 location and used the Mix Paste function of Adobe Audition CC 2017 to combine this file with every call sequence identified to the species level from our control sites in both 2017 and 2018 – a total of 15,403

files. These files were re-run through SonoBat using the program settings described above. A total of 50 files (approximately 0.3%) were misidentified or unidentifiable using the “1st” classifier, whereas SonoBat’s “SppAccp” classifier returned 1,056 mis- or unidentified sequences. Thus, we used the “1st” classifier in subsequent analyses. Given the extremely low error rate, it is unlikely that background noise could alter our inference from the generalized linear mixed effects models.

Bat foraging trials

We assessed foraging-modality switching in free-flying bats using an experimental paradigm that mimicked both terrestrial and aerial prey. We hypothesized that aerial prey would be preferred under higher ambient sound levels, which was more likely to mask the cues produced by walking and calling insects (**Fig S11**).

Prey-sound speaker playback

We standardized all prey-cue recordings to an amplitude of -0.1 dB, and all to a length of four minutes, with a sample rate of 48 kHz with 16-bit depth (WAV format). To create the field playbacks, we used Adobe Audition CC 2017 to create a one-hour file which linearly combined each of the cues in random order, such that each cue was played three times per hour without occurring twice in a row. A five-second crossfade was applied between each file transition, and a five-second fade at the beginning and end of each playback. We used weatherproof speakers (Eco Extreme, Grace Digital Inc., CA, USA) and an LS-7 player (Olympus, Shinjuku, Japan) powered by a LiFePO4 battery (AA Portable Power Corp, CA, USA) housed in waterproof

plastic containers to deliver the acoustic cues for passive listening bats in the field (**Fig S13**). Audio cues were looped continuously from dusk to dawn. Insect rustling sounds were less intense (52.1 dBF / 33.3 dBA) than cricket playback (86.4 dBF / 87.0 dBA), and the integrated hourly sound pressure level of prey playback speakers was 71.1 dBF / 71.6 dBA rel 20 µPa at 1 m in an anechoic room.

A note on treatment vs continuous analyses

There may be concerns that overall patterns in bird abundance or bat activity in relation to sound pressure levels may be driven by latent variables that are linked to river noise. For example, high sound level streams may be larger or higher sloped and thus may have different vegetation, relative humidity, or consequently communities of insect prey. Background frequency does not logically track linearly with these same variables (quiet and loud streams both may produce higher frequency measures for different reasons. Whitewater rivers can contain higher frequencies if they are intense, but slow-moving streams can as well due to leaves blowing in the wind). For this reason we focus here on sound pressure levels for the obvious connections to the above latent variables.

We first address logically why we do not believe this to be an issue, and then explore quantitative methods for addressing the potential concern.

Figure S6 shows nicely that all sites were strongly overlapping in sound levels (pre noise exposure), with the majority of the sound levels between 35 and 60 dB. When the experiment is turned "on", the majority of the energy in those experimental sites is now between 60 and 80 dB (right panel in Fig S6). Thus, nearly all of the high sound level sites came from the experiment. If there were confounding latent variables at the sites, we wouldn't expect to see such a strong signature of sound level in our analysis, since the streams that were experimentally made to have high sound levels originally had lower levels (Fig S6), thus the confounds of having larger streams and more humidity would not track linearly with sound levels – hence the power of the experiment. We do recognize that it is possible in control sites that the effects of sound level are correlated with a latent variable related to stream size, but those data are so much more sparse at the high end than the experimental data.

Additional confounding variables that almost certainly share some correlation to stream size and flow (and thus can absorb some of this variation in the model) are date (day of year), a quadratic version of date, riparian vegetation, and elevation – all of which were in our models, which help separate the effects of noise measures from other confounds. Thus, with this experimental design and analysis, we find it extremely unlikely that the effects would be due to a stream size confound.

There are also issues with designating these so called 'treatments'. We broadcast noise at some locations and not others. While this would seem, on the face of it, that there are clear designations of treatments, there certainly are not. We chose locations that were some distance from each noise playback area to create a continuum of sound pressure levels and background

frequencies. Thus, at many locations one can hear the river or shifted river broadcasted files, but nothing near the intensities at the playback locations themselves. So it appears that we have created continua of treatments – or *realized treatments*. We instead quantify the acoustic environment[25] and ask specifically what different components of noise (i.e. sound levels and frequency) do to drive wildlife populations and behavior, which also happen to help with the mechanistic understanding of the system. We would argue that a categorical analysis of a gradient is almost always going to be a more crude approach than a continuous one, and should only be used when continuous data are not available or when categories actually do not differ in the values that one would measure continuously.

One possible quantitative approach to alleviate any concerns are to include an interaction term between sound pressure levels and treatment in each of the bird and bat models. We cannot include this interaction here because the term is highly collinear. That is, the slope (estimate) of sound levels for control data is highly predictive of (and thus also predicted by) the slopes for the interaction of the treatments by sound level. In this situation, calculations regarding these predictors cannot be trusted, given such high variance inflation factor (VIF) scores for these parameters (Bird abundance: VIF = 23.7 for sound level by treatment interaction; Bat activity: VIF = 19.2 for sound level by treatment interaction; both of these scores are considered high by any assessment).

Thus, plotting model predicted lines (as we do in the main text; see Figures 2 and 3) are not necessarily going to reflect accurate estimates *when we include this interaction term*. For this

reason, it seems the only viable way to check inferences from experimental data line up with the overall inferences from the entire study is to create models that include only experimental data. At the top of Table S1, the first global bird model presented includes estimates for sound pressure level of -0.067 with a standard error of 0.018. The same model including only experimental sites (included at the bottom of Table S1) produces an estimate of -0.052 with a standard error of 0.019. Both of these estimates return p values below 0.01 and are overlapping estimates considering the standard errors presented here.

At the top of Table S5, the first global bat model presented includes estimates for sound pressure level of -0.082 with a standard error of 0.018. The same model including only experimental sites (included at the bottom of Table S5) produces an estimate of -0.097 with a standard error of 0.019. Both of these estimates return p values below 0.001 and are overlapping estimates considering the standard errors presented here.

We would conclude that it is very unlikely that estimates of how sound variables affect bird and bat abundance and activity are somehow confounded with stream properties at the control sites.

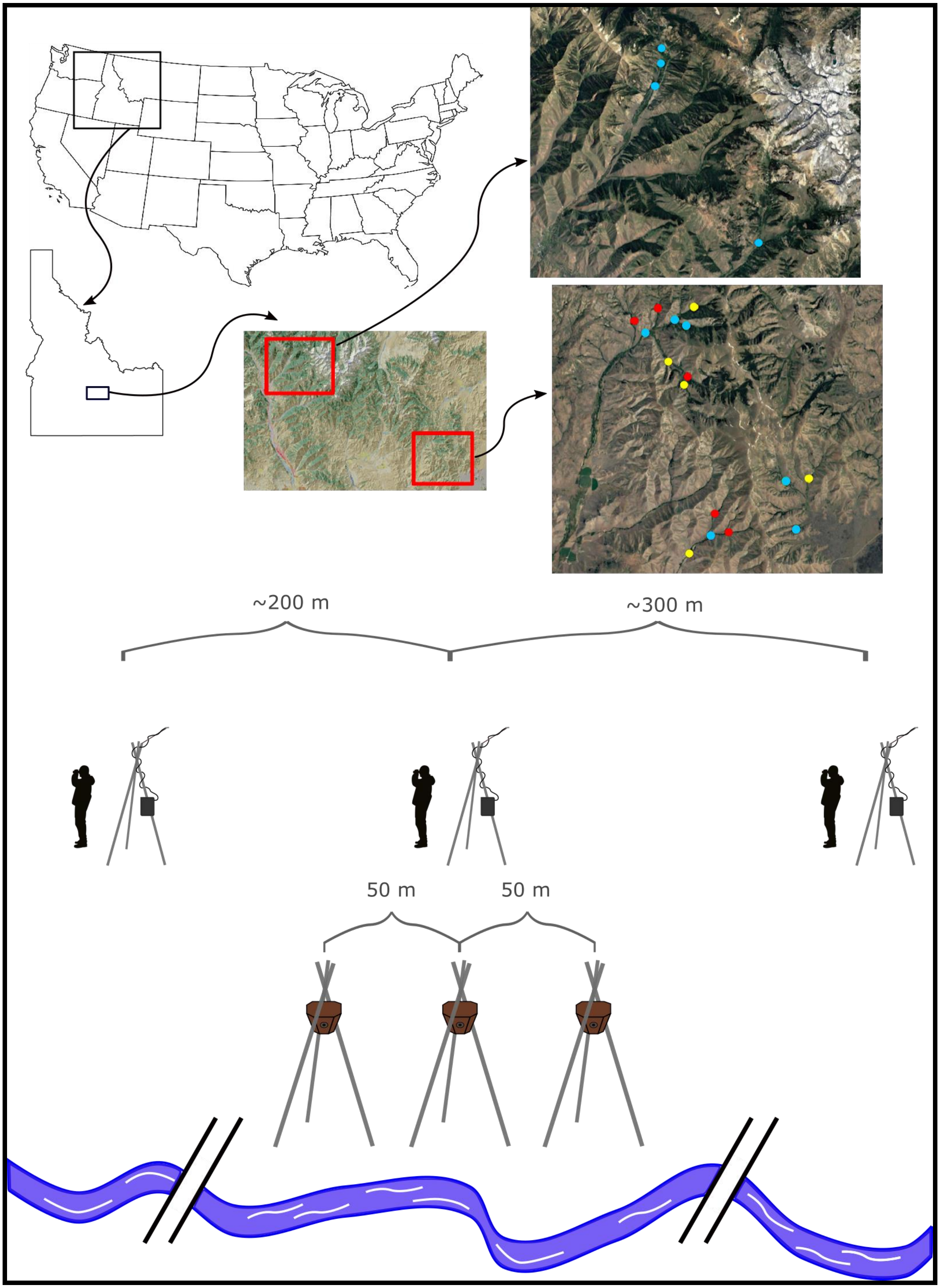
~200 m
~300 m
50 m
50 m

Fig S1.

Twenty stream sites in the Pioneer Mountains of Idaho were selected for the experiment (top). Blue dots represent control sites that naturally ranged in sound pressure levels and frequency. Yellow dots represent treatment sites that were exposed to experimental whitewater river noise. Red dots represent treatment sites that were exposed to spectrally-shifted whitewater river noise. At each site, three speaker towers (dummy speakers at controls) were set up 50 m apart in the riparian zone (layout not to scale). Three locations within each site (with a range of acoustic environments) were monitored for bird abundance (point counts) and bat activity (passive acoustic monitoring).

A

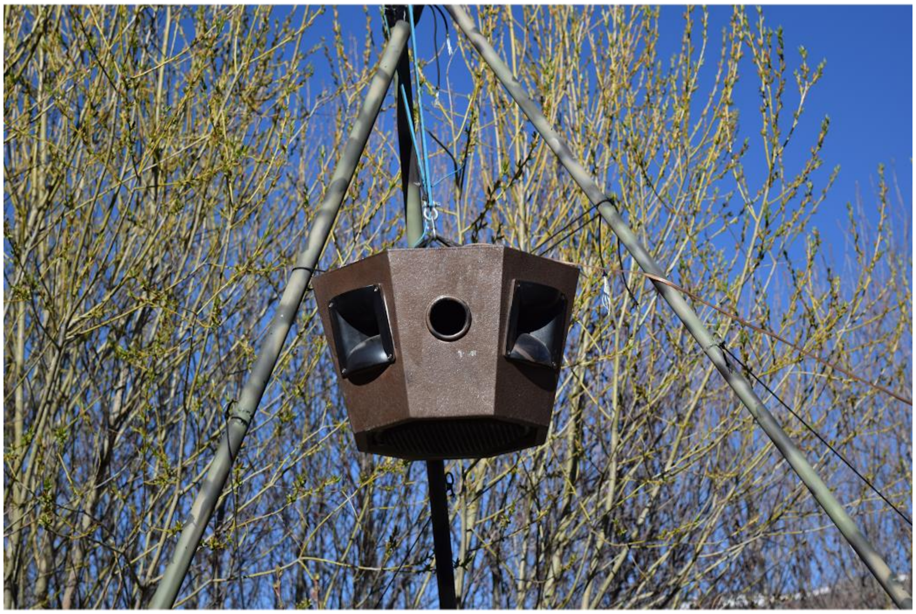

B

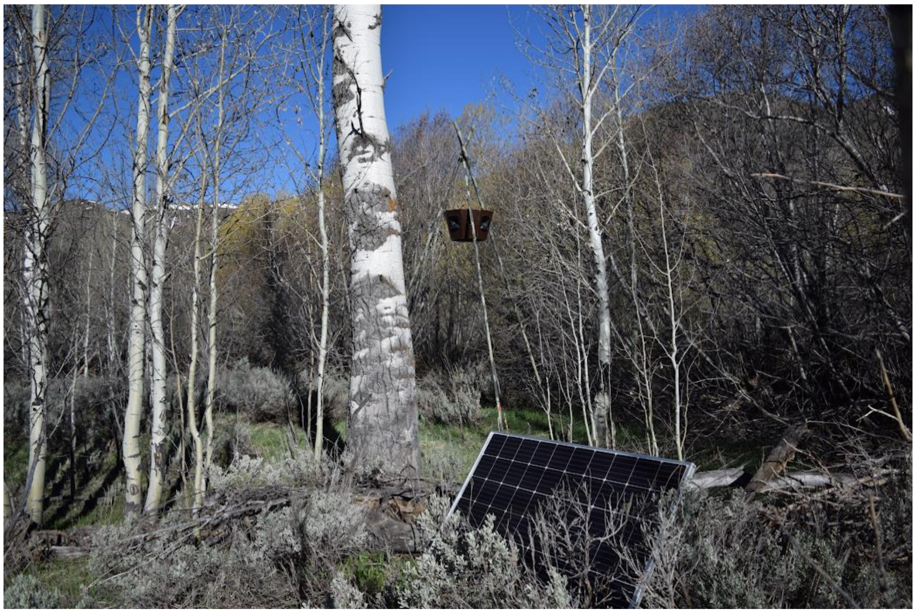

Fig S2.

Panel A: Octasound SP820A speaker broadcasting phantom river noise. Panel B: The same speaker, with solar panel, in the riparian area.

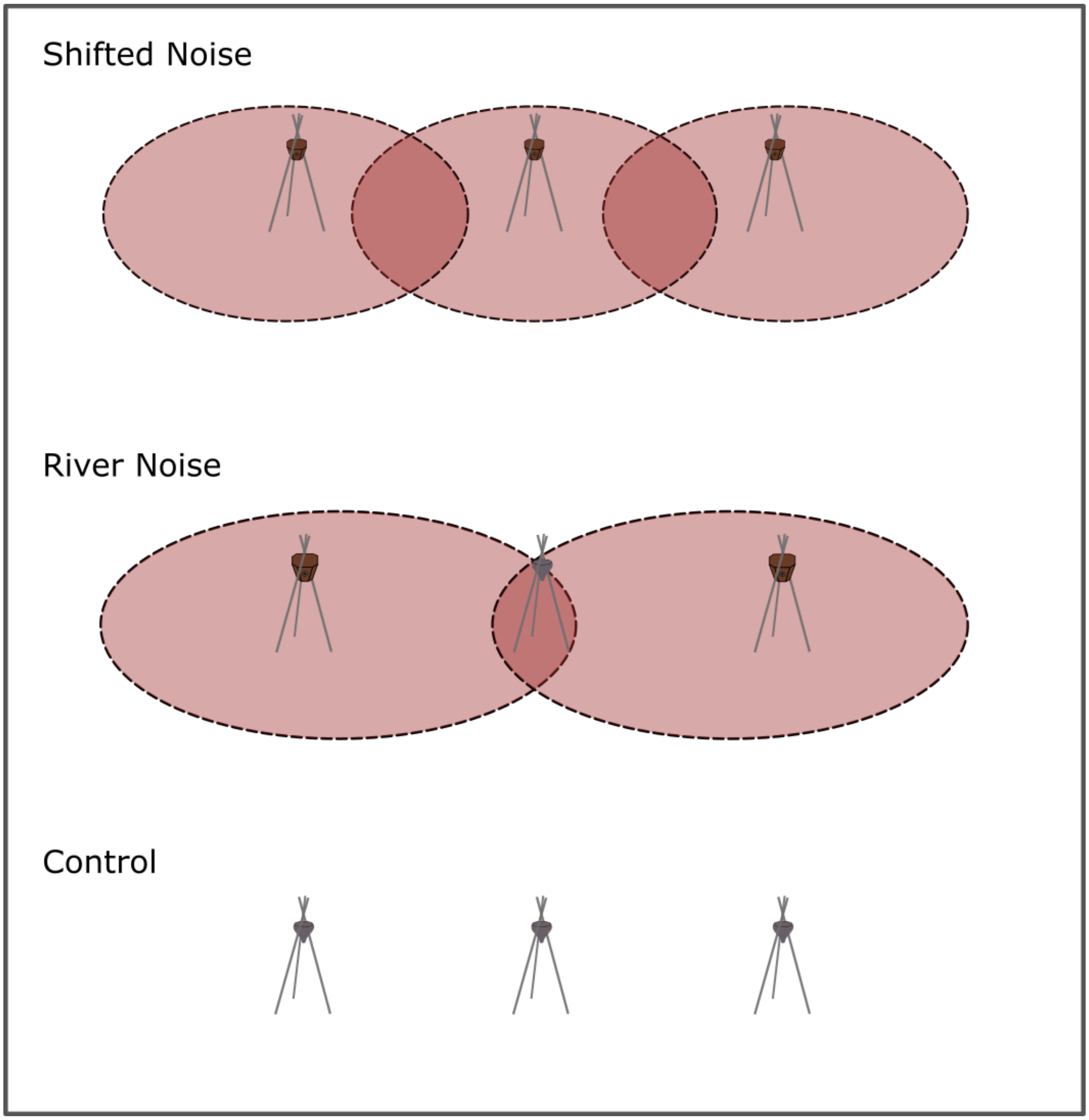

Fig S3.

Experimental site layout of the three types of sites. Red ovals are a heuristic depiction of sound exposure from speakers. ‘Dummy’ speakers are grey, with no ovals around them. Note that ‘Shifted’ noise was produced by three speakers that impacted slightly smaller areas, whereas ‘River’ noise was produced by two speakers that had a larger spatial footprint.

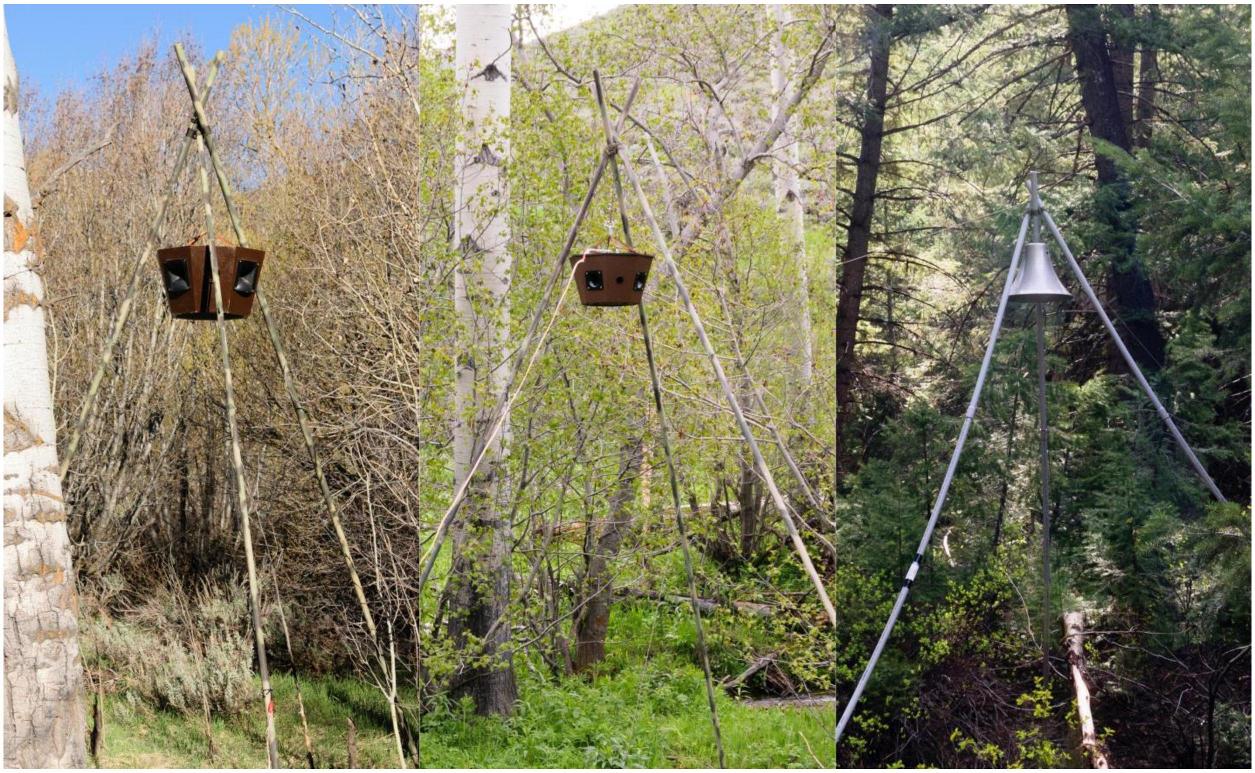

Fig S4.

Comparison of Octasound SP820A (left) and Octasound SP800A (center) speakers which broadcast phantom- and shifted-river noise, respectively, to a control ‘dummy’ speaker (right) that matched the approximate dimensions of the treatment speakers. Phantom and shifted speakers were suspended from metal conduit, while control speakers were suspended from PVC pipe. Each control speaker was paired with a dummy solar panel constructed from black particle board matching the dimensions of the treatment solar panels (not pictured).

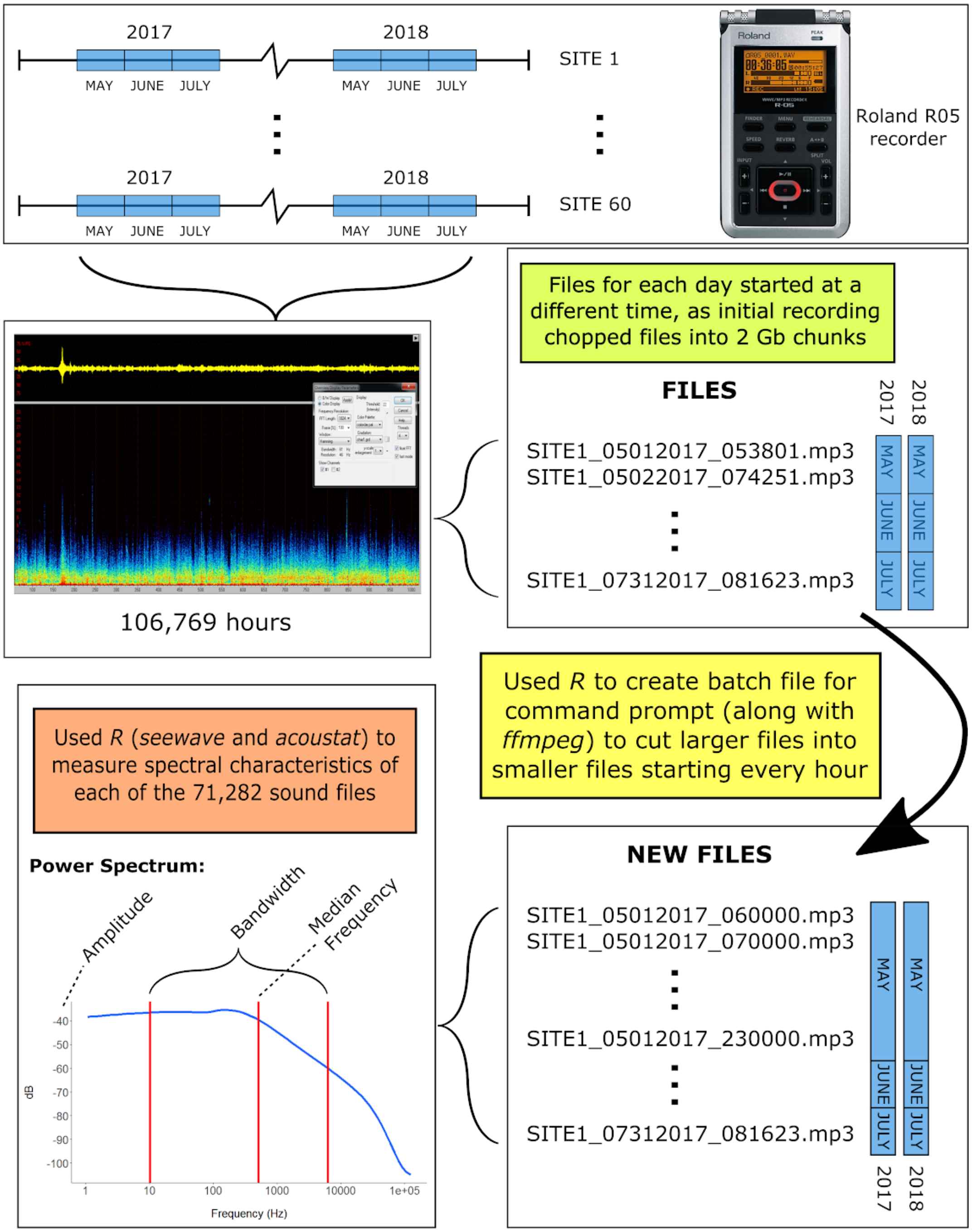

Fig S5.

Heuristic of scale and pipeline of acoustic environment quantification. Acoustic environments were continuously monitored at 60 locations, for two summers (2017 and 2018), totaling 100,000+ hours of recordings. Files were then analyzed by hour to obtain sound pressure level (not pictured) and median background frequency.

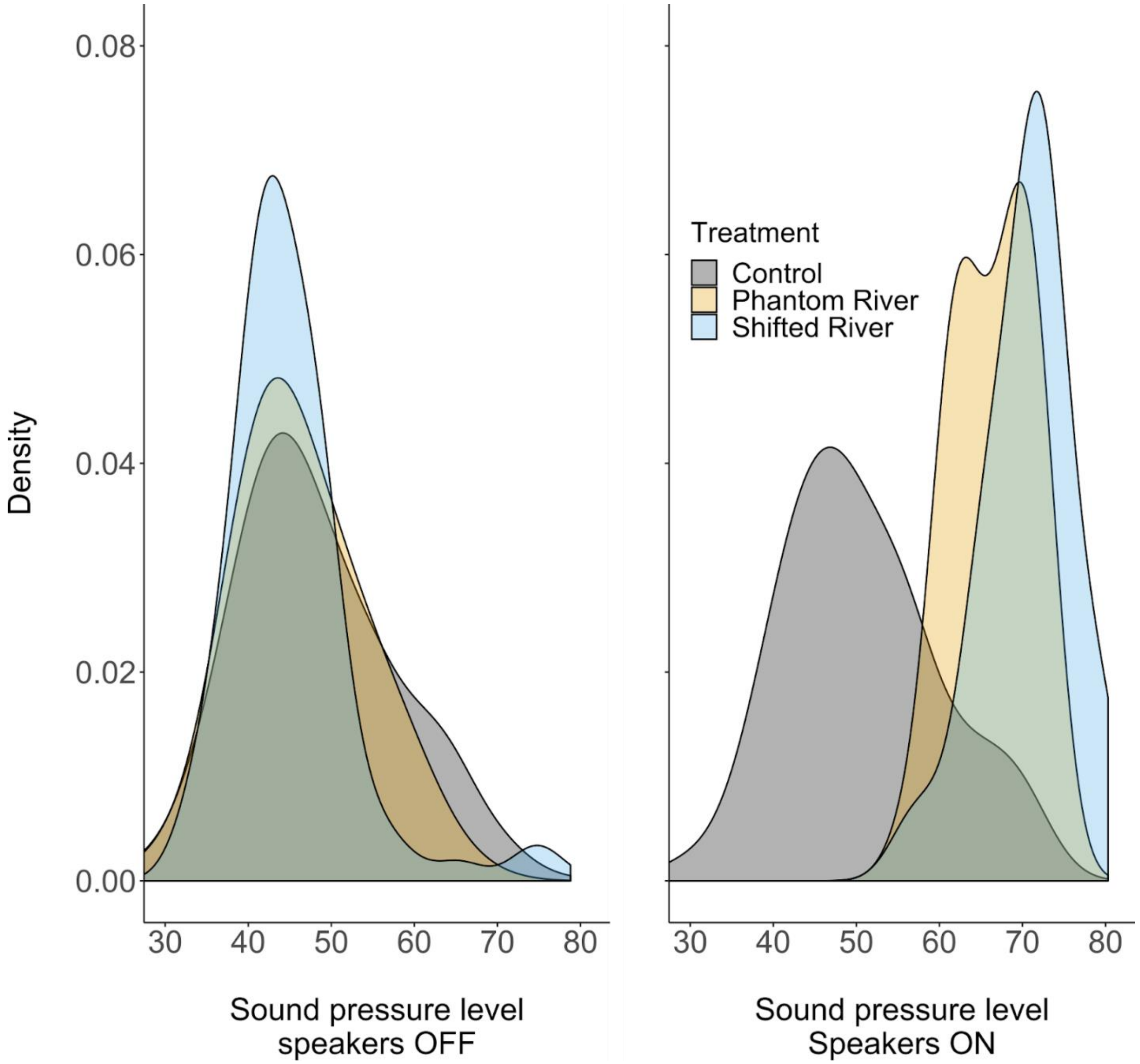

Fig S6.

The sound pressure levels of our sites showed little variation before broadcasting noise (left). However, when we experimentally added noise to 10 of these sites sound levels dramatically increased (right).

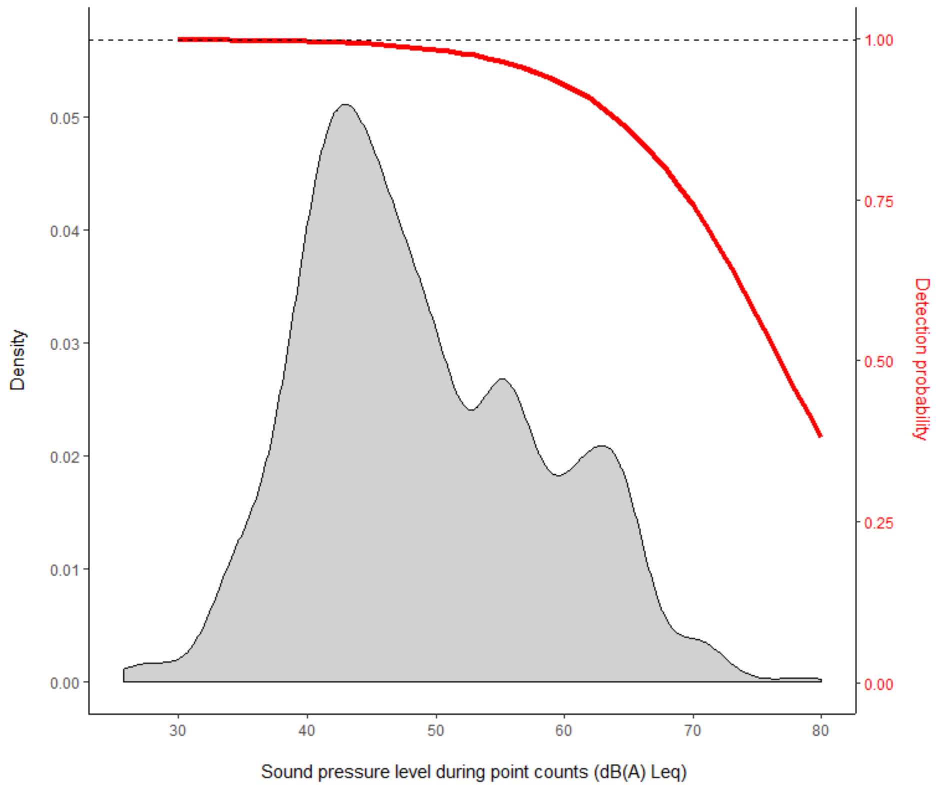

Fig S7.

The sound pressure level during point counts is shown in grey. Most counts fall below 55 dB(A), where the detection probability is calculated to be nearly 1. Detection probability (in red) is informed by a birdsong playback experiment with 6 individual observers.

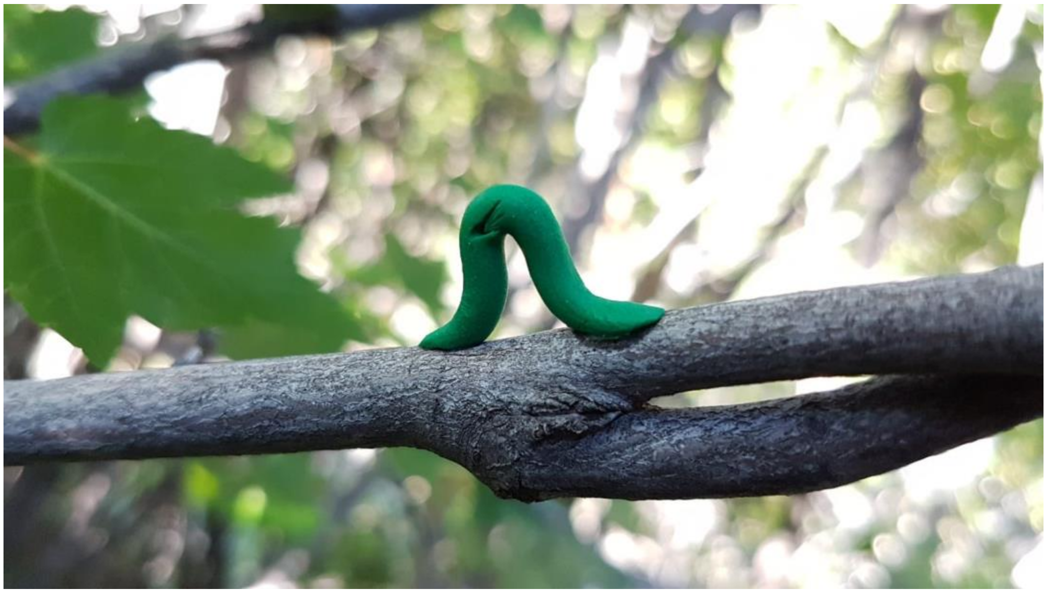

Fig S8.

Clay caterpillar glued to a branch in the riparian area to test foraging rates of birds in various acoustic environments. Note the bird beak mark in the upper portion of the caterpillar.

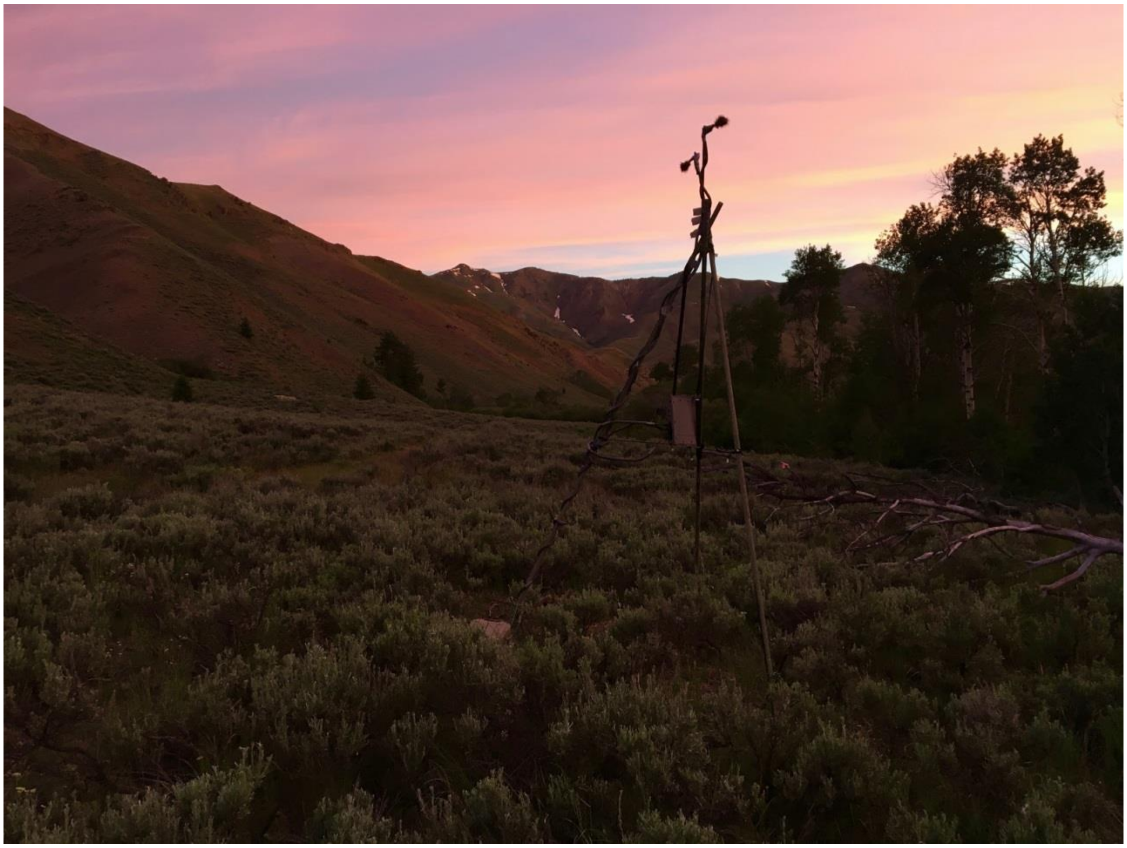

Fig S9.

Bat detector (Wildlife Acoustics Song Meter 3) at a monitoring location near sunset. Bat detector was fixed to a tripod made out of ~3 m long metal conduit.

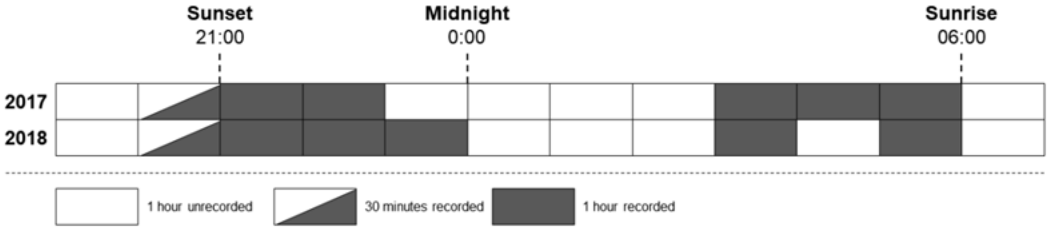

Fig S10.

Recording schedules for bat detector (Wildlife acoustics SM3) during the 2017 and 2018 field seasons slightly differ due to other experiments that occurred in tandem.

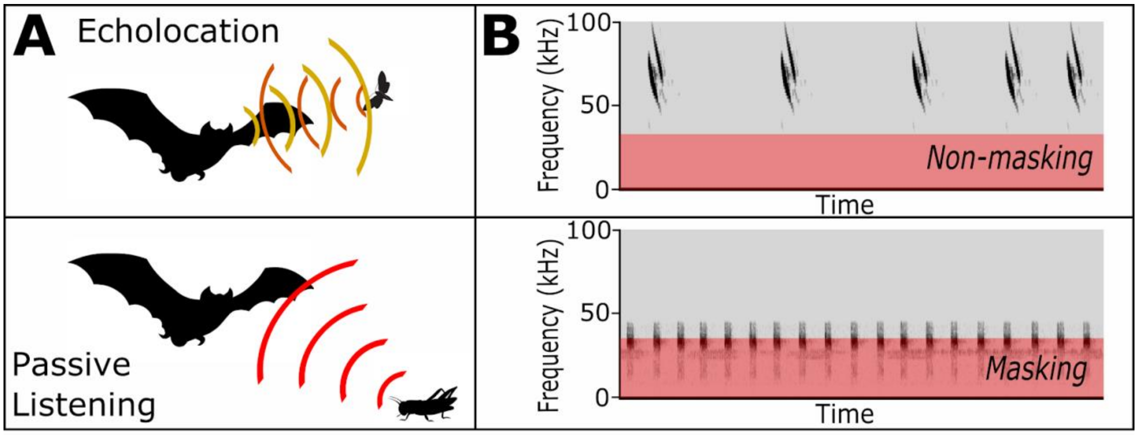

Fig S11.

A) Depiction of two foraging styles, aerial hawking via echolocation (active listening) in the top and passive listening (to prey generated sounds) in the bottom. B) We predict, based on spectral overlap, that aerial hawking tasks would not be masked, whereas passive listening would be.

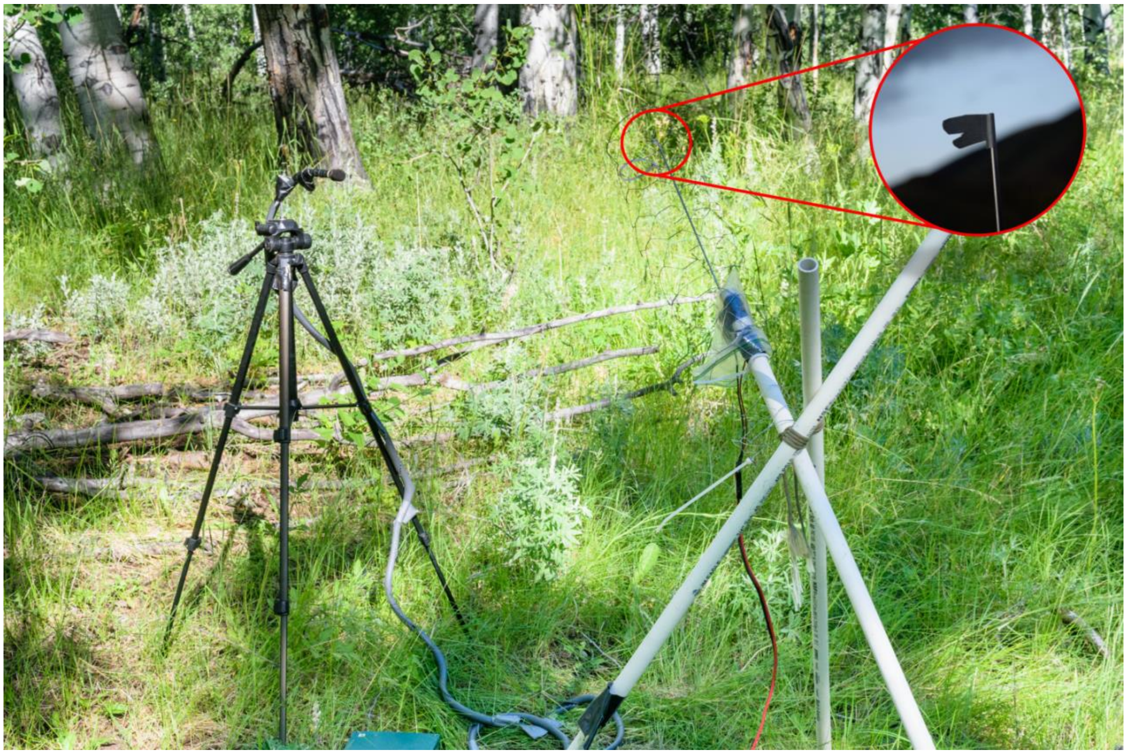

Fig S12.

Robotic insect wing (pictured in inset) was used to test foraging strategy switching in flexible bats.

Ultrasonic microphone on a tripod, with Wildlife Acoustics SM2 recording unit in bottom of photo.

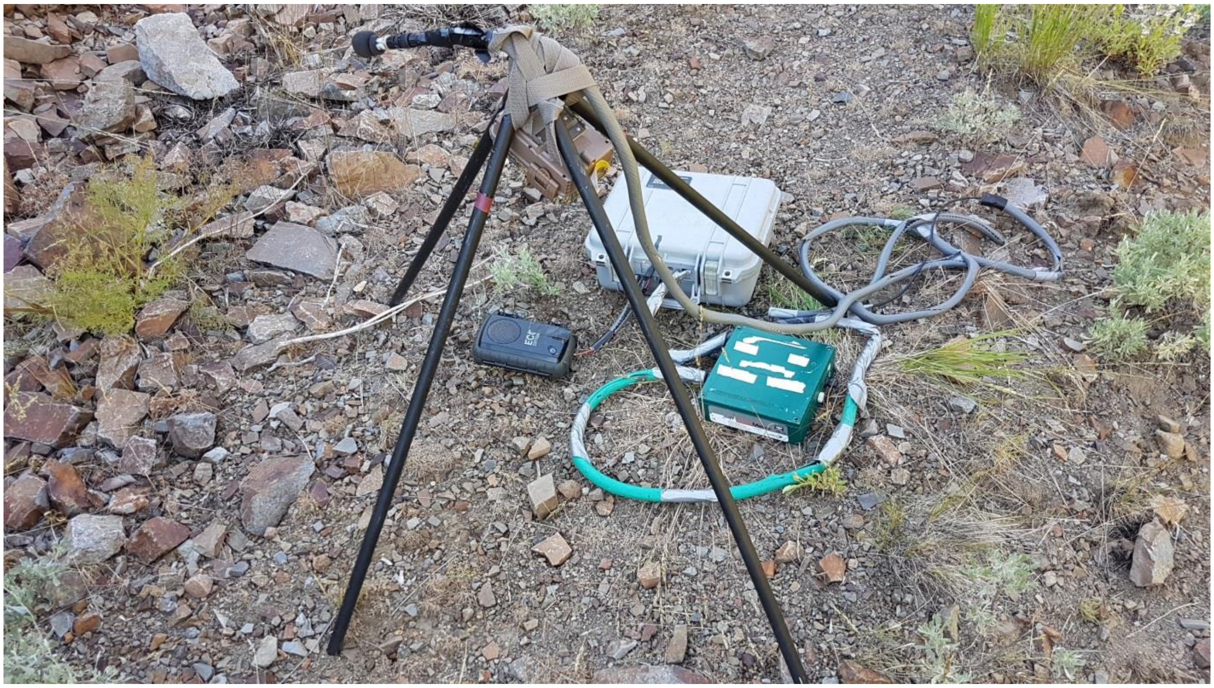

Fig S13.

Insect prey-sound speaker (small black box) to test foraging strategy switching in flexible bats. Ultrasonic microphone and wildlife acoustics SM2 recording unit (green box), powered by Lithium batteries (grey box) also pictured.

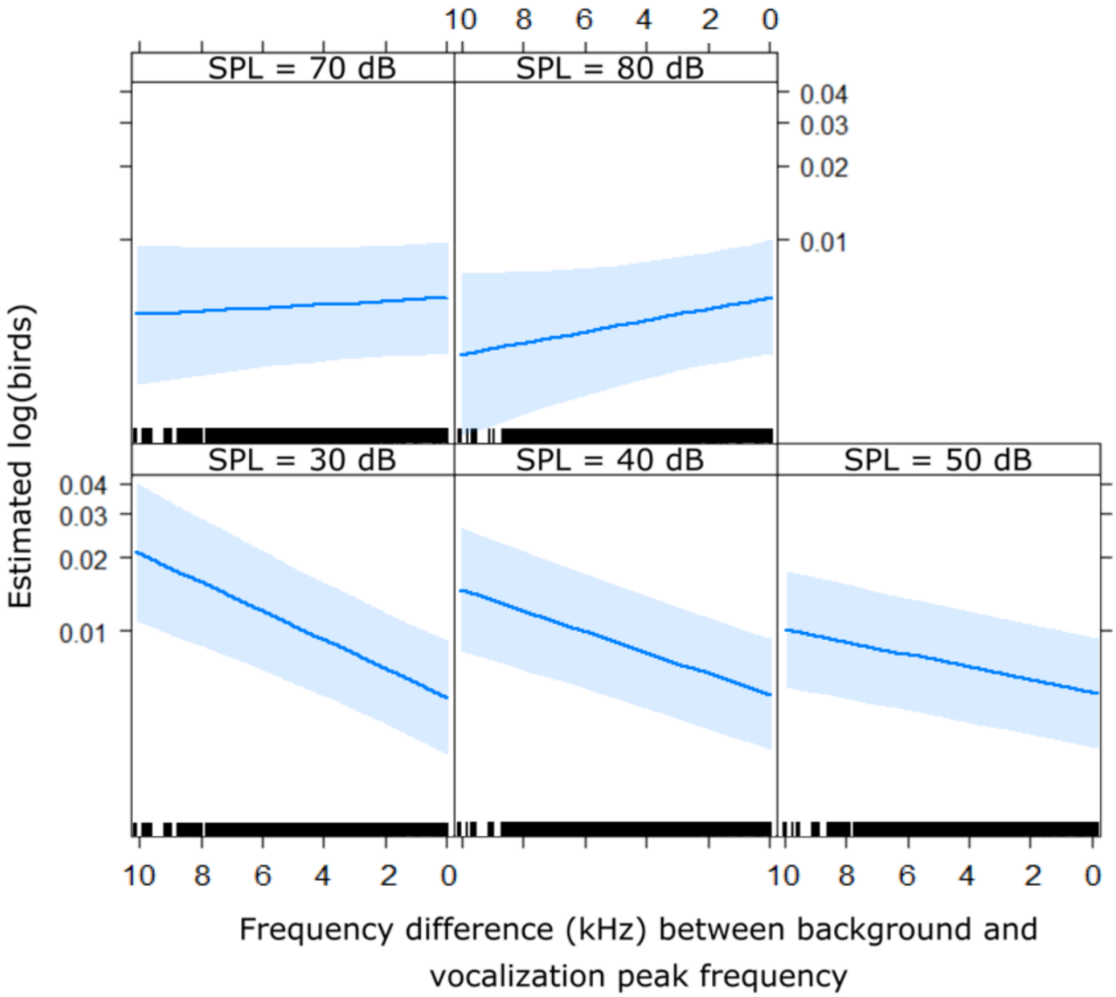

Fig S14.

Effects of frequency difference across a range of sound pressure levels (SPL). When sound levels are low (e.g. 30 dB), the amount of spectral overlap is important in determining bird abundance. That is, the higher the difference in frequency was between the background acoustic environment and the peak frequency of a given species' vocalization, the higher the estimated count of birds would be (high negative slopes within these flipped x-axis plots). Yet, when sound pressure level was high (e.g. 70 and 80 dB), the effect of frequency difference was negligible (flat lines in top two panels). Lines in each panel are the estimated marginal means (predicted lines across the range of frequency difference while holding all other covariates constant at their mean values, except for sound pressure level which is varied in each panel). Shaded areas are 95% confidence intervals.

Table S1.

Bird abundance model summary tables. All continuous variables are centered to the mean, and standardized to one standard deviation. Abundance data are analyzed with a negative binomial distribution (log link).

Model: Global	Noise control: Observer experiment				
Variable	Estimate	SE	*CI*	z value	*p*
Intercept	-4.978	0.263	-5.492 – -4.460	-18.959	<0.001
Sound pressure (SPL)	-0.067	0.018	-0.102 – -0.030	-3.764	<0.001
Background freq. (BF)	-0.031	0.021	-0.072 – 0.010	-1.480	0.139
Spectral overlap (SO)	0.066	0.016	0.034 – 0.100	4.031	<0.001
Riparian vegetation	0.127	0.026	0.077 – 0.180	4.917	<0.001
Elevation	0.113	0.085	-0.053 – 0.280	1.331	0.183
Ordinal date	0.044	0.017	0.012 – 0.080	2.671	0.008
Ordinal date^2	-0.107	0.018	-0.143 – -0.070	-5.776	<0.001
Year	0.253	0.037	0.180 – 0.330	6.765	<0.001
SPL:BF	0.033	0.018	-0.003 – 0.070	1.817	0.069
SPL:SO	-0.057	0.016	-0.088 – -0.030	-3.549	<0.001

Model: Global	Noise control: Noise removal model				
Variable	Estimate	SE	*CI*	z value	*p*
Intercept	-5.007	0.265	-5.527 – -4.490	-18.895	<0.001
Sound pressure (SPL)	-0.069	0.018	-0.103 – -0.030	-3.863	<0.001
Background freq. (BF)	-0.032	0.021	-0.073 – 0.010	-1.554	0.120
Spectral overlap (SO)	0.066	0.016	0.034 – 0.100	4.049	<0.001
Riparian vegetation	0.131	0.026	0.080 – 0.180	5.038	<0.001
Elevation	0.118	0.084	-0.047 – 0.280	1.404	0.160
Ordinal date	0.256	0.037	0.183 – 0.330	6.837	<0.001
Ordinal date^2	0.047	0.017	0.015 – 0.080	2.844	0.004
Year	-0.108	0.018	-0.144 – -0.070	-5.84	<0.001
SPL:BF	0.034	0.018	-0.002 – 0.070	1.859	0.063
SPL:SO	-0.056	0.016	-0.087 – -0.020	-3.477	0.001

Model: Visual counts	Noise control: Observer ear plugs				
Variable	Estimate	SE	*CI*	z value	*p*
Intercept	-5.755	0.281	-6.305 – -5.200	-20.49	<0.001
Sound pressure (SPL)	-0.139	0.043	-0.223 – -0.050	-3.212	0.001
Background freq. (BF)	0.009	0.044	-0.077 – 0.090	0.208	0.835
Spectral overlap (SO)	0.102	0.034	0.036 – 0.170	3.027	0.002
Riparian vegetation	0.150	0.053	0.047 – 0.250	2.844	0.004
Elevation	-0.222	0.094	-0.405 – -0.040	-2.369	0.018
Ordinal date	0.037	0.037	-0.034 – 0.110	1.019	0.308

Ordinal date^2	-0.060	0.041	-0.140 – 0.020	-1.459	0.145
Year	0.039	0.083	-0.125 – 0.200	0.464	0.642
SPL:BF	0.148	0.040	0.069 – 0.230	3.689	<0.001
SPL:SO	-0.012	0.033	-0.078 – 0.050	-0.367	0.714

Model: Control sites	Noise control: Observer experiment				
Variable	Estimate	SE	*CI*	z value	*p*
Intercept	-5.206	0.296	-5.786 – -4.630	-17.596	<0.001
Sound pressure (SPL)	-0.123	0.058	-0.237 – -0.010	-2.117	0.034
Background freq. (BF)	-0.095	0.042	-0.178 – -0.010	-2.236	0.025
Spectral overlap (SO)	0.062	0.030	0.004 – 0.120	2.094	0.036
Riparian vegetation	-0.04	0.051	-0.140 – 0.060	-0.778	0.436
Elevation	-0.301	0.111	-0.519 – -0.080	-2.704	0.007
Ordinal date	0.034	0.027	-0.019 – 0.090	1.255	0.210
Ordinal date^2	-0.105	0.029	-0.162 – -0.050	-3.582	<0.001
Year	0.215	0.058	0.100 – 0.330	3.670	<0.001
SPL:BF	0.023	0.045	-0.065 – 0.110	0.512	0.608
SPL:SO	-0.146	0.032	-0.209 – -0.080	-4.502	<0.001

Model: Experimental sites only	Noise control: Observer experiment				
Variable	Estimate	SE	*CI*	z value	*p*
Intercept	-5.01	0.3	-5.597 - -4.42	-16.716	<0.001
Sound pressure (SPL)	-0.052	0.02	-0.09 - -0.01	-2.668	0.008
Background freq. (BF)	-0.008	0.026	-0.059 - 0.04	-0.304	0.761
Spectral overlap (SO)	0.04	0.021	-0.001 - 0.08	1.898	0.058
Riparian vegetation	0.129	0.022	0.085 - 0.17	5.735	<0.001
Elevation	0.159	0.129	-0.094 - 0.41	1.233	0.218
Ordinal date	0.057	0.021	0.016 - 0.10	2.714	0.007
Ordinal date^2	-0.104	0.024	-0.151 - -0.06	-4.272	<0.001
Year	0.29	0.05	0.192 - 0.39	5.824	<0.001
SPL:BF	-0.008	0.022	-0.051 - 0.03	-0.378	0.706
SPL:SO	0.036	0.023	-0.008 - 0.08	1.587	0.113

Table S2.

Individual bird species models. All continuous variables are centered to the mean, and standardized to one standard deviation. Abundance data are analyzed with a negative binomial distribution (log link).

	Common		Latin		
	American crow		*Corvus brachyrhynchos*		
Predictors	Estimates	SE	*CI*	Z	*p*
Intercept	-5.57	0.75	-4.10 – -7.04	-7.44	<0.01
SPL	0.26	0.31	0.87 – -0.35	0.83	0.41
Background Freq	-0.72	1.02	1.28 – -2.72	-0.7	0.48
Spectral Overlap	0.57	1.10	2.73 – -1.59	0.52	0.60
Vegetation	0.19	0.29	0.76 – -0.38	0.65	0.51
Elevation	-1.71	0.57	-0.59 – -2.83	-2.97	<0.01
Ordinal date	-0.30	0.24	0.17 – -0.77	-1.22	0.22
Ordinal date^2	0.08	0.26	0.59 – -0.43	0.32	0.75
Year	0.08	0.58	1.22 – -1.06	0.14	0.89
SPL:Background Freq	1.03	1.78	4.52 – -2.46	0.58	0.56
SPL:Spectral Overlap	-0.83	1.83	2.76 – -4.42	-0.45	0.65

	Common		Latin		
	American Kestrel		*Falco sparverius*		
Predictors	Estimates	SE	*CI*	Z	*p*
Intercept	-5.05	0.51	-4.05 – -6.05	-9.98	<0.01
SPL	-0.01	0.26	0.50 – -0.52	-0.05	0.96
Background Freq	0.37	0.22	0.80 – -0.06	1.69	0.09
Spectral Overlap	-0.12	0.18	0.23 – -0.47	-0.68	0.49
Vegetation	-0.06	0.25	0.43 – -0.55	-0.24	0.81
Elevation	-0.81	0.34	-0.14 – -1.48	-2.40	0.02
Ordinal date	-0.55	0.22	-0.12 – -0.98	-2.56	0.01
Ordinal date^2	0.71	0.23	1.16 – 0.26	3.10	<0.01
Year	-1.09	0.57	0.03 – -2.21	-1.92	0.05
SPL:Background Freq	<0.00	0.21	0.41 – -0.41	-0.02	0.98
SPL:Spectral Overlap	-0.11	0.20	0.28 – -0.50	-0.56	0.58

	Common		Latin		
	American robin		*Turdus migratorius*		
Predictors	Estimates	SE	*CI*	Z	*p*
Intercept	-2.70	0.26	-2.19 – -3.21	-10.56	<0.01
SPL	0.01	0.09	0.19 – -0.17	0.13	0.90

Background Freq	-0.18	0.20	0.21 – -0.57	-0.92	0.36
Spectral Overlap	0.33	0.18	0.68 – -0.02	1.86	0.06
Vegetation	0.13	0.13	0.38 – -0.12	0.99	0.32
Elevation	-0.03	0.21	0.38 – -0.44	-0.17	0.87
Ordinal date	-0.19	0.09	-0.01 – -0.37	-2.14	0.03
Ordinal date^2	-0.13	0.09	0.05 – -0.31	-1.43	0.15
Year	0.09	0.19	0.46 – -0.28	0.47	0.64
SPL:Background Freq	-0.29	0.22	0.14 – -0.72	-1.32	0.19
SPL:Spectral Overlap	0.25	0.21	0.66 – -0.16	1.21	0.22

	Common		Latin		
	Black-capped Chickadee		*Poecile atricapillus*		
Predictors	Estimates	SE	*CI*	Z	*p*
Intercept	-6.14	0.83	-4.51 – -7.77	-7.42	<0.01
SPL	-0.08	0.18	0.27 – -0.43	-0.43	0.67
Background Freq	0.04	0.22	0.47 – -0.39	0.17	0.87
Spectral Overlap	0.25	0.20	0.64 – -0.14	1.23	0.22
Vegetation	-0.13	0.30	0.46 – -0.72	-0.43	0.67
Elevation	0.56	0.48	1.50 – -0.38	1.15	0.25
Ordinal date	-0.16	0.18	0.19 – -0.51	-0.93	0.35
Ordinal date^2	0.85	0.21	1.26 – 0.44	4.11	<0.01
Year	0.02	0.46	0.92 – -0.88	0.05	0.96
SPL:Background Freq	0.01	0.20	0.40 – -0.38	0.05	0.96
SPL:Spectral Overlap	-0.13	0.22	0.30 – -0.56	-0.61	0.54

	Common		Latin		
	Brown-headed cowbird		*Molothrus ater*		
Predictors	Estimates	SE	*CI*	Z	*p*
Intercept	-4.89	0.45	-4.01 – -5.77	-10.77	<0.01
SPL	-0.34	0.26	0.17 – -0.85	-1.29	0.20
Background Freq	-0.14	0.49	0.82 – -1.10	-0.29	0.77
Spectral Overlap	0.19	0.43	1.03 – -0.65	0.44	0.66
Vegetation	0.41	0.18	0.76 – 0.06	2.22	0.03
Elevation	-1.26	0.25	-0.77 – -1.75	-5.12	<0.01
Ordinal date	0.05	0.21	0.46 – -0.36	0.22	0.83
Ordinal date^2	-0.39	0.24	0.08 – -0.86	-1.64	0.10
Year	0.66	0.49	1.62 – -0.30	1.35	0.18
SPL:Background Freq	-0.07	0.54	0.99 – -1.13	-0.13	0.90
SPL:Spectral Overlap	-0.60	0.51	0.40 – -1.60	-1.19	0.24

	Common		Latin		

	Brewer's Sparrow		*Spizella breweri*		
Predictors	Estimates	SE	*CI*	Z	*p*
Intercept	-10.1	1.81	-6.55 – -13.65	-5.59	<0.01
SPL	0.77	0.26	1.28 – 0.26	3.01	<0.01
Background Freq	-0.39	0.34	0.28 – -1.06	-1.17	0.24
Spectral Overlap	-0.01	0.31	0.60 – -0.62	-0.04	0.97
Vegetation	-1.3	0.58	-0.16 – -2.44	-2.23	0.03
Elevation	-3.25	1.03	-1.23 – -5.27	-3.15	<0.01
Ordinal date	-0.90	0.36	-0.19 – -1.61	-2.53	0.01
Ordinal date^2	-0.11	0.27	0.42 – -0.64	-0.40	0.69
Year	0.90	0.62	2.12 – -0.32	1.45	0.15
SPL:Background Freq	-0.14	0.25	0.35 – -0.63	-0.54	0.59
SPL:Spectral Overlap	0.84	0.28	1.39 – 0.29	2.95	<0.01

	Common		Latin		
	Bullock's Oriole		*Icterus bullockii*		
	Estimates	SE	*CI*	Z	*p*
Intercept	-5.62	0.67	-4.31 – -6.93	-8.35	<0.01
SPL	0.33	0.36	1.04 – -0.38	0.93	0.35
Background Freq	0.54	0.60	1.72 – -0.64	0.91	0.36
Spectral Overlap	-0.12	0.42	0.70 – -0.94	-0.29	0.77
Vegetation	0.26	0.29	0.83 – -0.31	0.89	0.38
Elevation	-0.91	0.45	-0.03 – -1.79	-2.01	0.04
Ordinal date	0.23	0.19	0.60 – -0.14	1.18	0.24
Ordinal date^2	-0.75	0.21	-0.34 – -1.16	-3.63	<0.01
Year	1.08	0.41	1.88 – 0.28	2.62	0.01
SPL:Background Freq	-0.50	0.69	0.85 – -1.85	-0.72	0.47
SPL:Spectral Overlap	0.03	0.50	1.01 – -0.95	0.06	0.95

	Common		Latin		
	Chipping Sparrow		*Spizella passerina*		
Predictors	Estimates	SE	*CI*	Z	*p*
Intercept	-5.11	0.65	-3.84 – -6.38	-7.86	<0.01
SPL	-0.11	0.37	0.62 – -0.84	-0.31	0.76
Background Freq	-1.95	2.18	2.32 – -6.22	-0.89	0.37
Spectral Overlap	-1.19	1.62	1.99 – -4.37	-0.73	0.46
Vegetation	-0.55	0.31	0.06 – -1.16	-1.77	0.08
Elevation	0.39	0.32	1.02 – -0.24	1.22	0.22
Ordinal date	0.18	0.20	0.57 – -0.21	0.87	0.38
Ordinal date^2	0.31	0.21	0.72 – -0.10	1.50	0.13
Year	-0.54	0.44	0.32 – -1.40	-1.23	0.22

SPL:Background Freq	1.56	1.55	4.60 – -1.48	1.01	0.31
SPL:Spectral Overlap	0.88	1.16	3.15 – -1.39	0.76	0.45

	Common		Latin		
	Dark-eyed Junco		*Junco hyemalis*		
Predictors	Estimates	SE	*CI*	Z	*p*
Intercept	-4.85	0.49	-3.89 – -5.81	-9.83	<0.01
SPL	0.03	0.19	0.40 – -0.34	0.16	0.88
Background Freq	-0.52	0.62	0.70 – -1.74	-0.83	0.41
Spectral Overlap	-0.60	0.49	0.36 – -1.56	-1.23	0.22
Vegetation	-0.21	0.22	0.22 – -0.64	-0.95	0.34
Elevation	0.42	0.32	1.05 – -0.21	1.31	0.19
Ordinal date	-0.49	0.20	-0.10 – -0.88	-2.51	0.01
Ordinal date^2	0.09	0.19	0.46 – -0.28	0.49	0.62
Year	-0.14	0.42	0.68 – -0.96	-0.33	0.74
SPL:Background Freq	0.42	0.63	1.65 – -0.81	0.68	0.50
SPL:Spectral Overlap	0.42	0.49	1.38 – -0.54	0.85	0.39

	Common		Latin		
	Dusky Flycatcher		*Empidonax oberholseri*		
Predictors	Estimates	SE	*CI*	Z	*p*
Intercept	-3.80	0.26	-3.29 – -4.31	-14.49	<0.01
SPL	-0.39	0.14	-0.12 – -0.66	-2.83	<0.01
Background Freq	-0.07	0.21	0.34 – -0.48	-0.36	0.72
Spectral Overlap	0.07	0.18	0.42 – -0.28	0.42	0.67
Vegetation	-0.11	0.14	0.16 – -0.38	-0.78	0.43
Elevation	0.58	0.17	0.91 – 0.25	3.44	<0.01
Ordinal date	0.05	0.12	0.29 – -0.19	0.42	0.68
Ordinal date^2	-0.28	0.13	-0.03 – -0.53	-2.12	0.03
Year	0.73	0.27	1.26 – 0.20	2.68	0.01
SPL:Background Freq	-0.13	0.22	0.30 – -0.56	-0.62	0.54
SPL:Spectral Overlap	0.22	0.18	0.57 – -0.13	1.22	0.22

	Common		Latin		
	European starling		*Sturnus vulgaris*		
Predictors	Estimates	SE	*CI*	Z	*p*
Intercept	-7.11	1.46	-4.25 – -9.97	-4.88	<0.01
SPL	-0.32	0.63	0.91 – -1.55	-0.51	0.61
Background Freq	-0.05	1.28	2.46 – -2.56	-0.04	0.97
Spectral Overlap	-0.51	0.88	1.21 – -2.23	-0.58	0.56

Vegetation	-0.33	0.45	0.55 – -1.21	-0.73	0.46
Elevation	-1.06	0.59	0.10 – -2.22	-1.81	0.07
Ordinal date	-3.23	1.50	-0.29 – -6.17	-2.15	0.03
Ordinal date^2	-1.88	0.89	-0.14 – -3.62	-2.12	0.03
Year	1.04	1.21	3.41 – -1.33	0.86	0.39
SPL:Background Freq	-1.15	1.22	1.24 – -3.54	-0.95	0.34
SPL:Spectral Overlap	-0.51	0.76	0.98 – -2.00	-0.68	0.50

	Common		Latin		
	House wren		*Troglodytes aedon*		
Predictors	Estimates	SE	*CI*	Z	*p*
Intercept	-2.09	0.35	-1.4 – -2.78	-5.96	<0.01
SPL	-0.02	0.06	0.1 – -0.14	-0.33	0.74
Background Freq	0.19	0.07	0.33 – 0.05	2.9	<0.01
Spectral Overlap	-0.07	0.05	0.03 – -0.17	-1.4	0.16
Vegetation	0.53	0.10	0.73 – 0.33	5.42	<0.01
Elevation	0.33	0.29	0.9 – -0.24	1.13	0.26
Ordinal date	-0.24	0.05	-0.14 – -0.34	-4.34	<0.01
Ordinal date^2	-0.07	0.06	0.05 – -0.19	-1.23	0.22
Year	0.16	0.12	0.4 – -0.08	1.35	0.18
SPL:Background Freq	-0.08	0.06	0.04 – -0.2	-1.3	0.19
SPL:Spectral Overlap	-0.11	0.05	-0.01 – -0.21	-2.33	0.02

	Common		Latin		
	Lazuli Bunting		*Passerina amoena*		
Predictors	Estimates	SE	*CI*	Z	*p*
Intercept	-1.77	0.18	-1.42 – -2.12	-9.84	<0.01
SPL	0.08	0.06	0.20 – -0.04	1.37	0.17
Background Freq	-0.16	0.08	0.00 – -0.32	-1.97	0.05
Spectral Overlap	-0.16	0.07	-0.02 – -0.30	-2.36	0.02
Vegetation	-0.01	0.08	0.15 – -0.17	-0.13	0.90
Elevation	-0.32	0.15	-0.03 – -0.61	-2.12	0.03
Ordinal date	1.34	0.10	1.54 – 1.14	13.79	<0.01
Ordinal date^2	-0.71	0.08	-0.55 – -0.87	-8.54	<0.01
Year	0.40	0.11	0.62 – 0.18	3.67	<0.01
SPL:Background Freq	-0.07	0.07	0.07 – -0.21	-0.95	0.34
SPL:Spectral Overlap	-0.01	0.07	0.13 – -0.15	-0.14	0.89

	Common		Latin		
	MacGillivray's Warbler		*Geothlypis tolmiei*		

Predictors	Estimates	SE	*CI*	Z	*p*
Intercept	-2.53	0.25	-2.04 – -3.02	-10.13	<0.01
SPL	0.12	0.08	0.28 – -0.04	1.55	0.12
Background Freq	-0.53	0.15	-0.24 – -0.82	-3.53	<0.01
Spectral Overlap	-0.08	0.11	0.14 – -0.30	-0.75	0.45
Vegetation	0.08	0.12	0.32 – -0.16	0.69	0.49
Elevation	0.21	0.20	0.6 – -0.18	1.04	0.30
Ordinal date	0.71	0.11	0.93 – 0.49	6.69	<0.01
Ordinal date^2	-0.79	0.11	-0.57 – -1.01	-7.01	<0.01
Year	0.53	0.16	0.84 – 0.22	3.29	<0.01
SPL:Background Freq	0.40	0.11	0.62 – 0.18	3.62	<0.01
SPL:Spectral Overlap	0.15	0.10	0.35 – -0.05	1.54	0.12

Common	Latin
Northern flicker	*Colaptes auratus*

Predictors	Estimates	SE	*CI*	Z	*p*
Intercept	-3.75	0.29	-3.18 – -4.32	-12.76	<0.01
SPL	-0.16	0.15	0.13 – -0.45	-1.06	0.29
Background Freq	-0.01	0.25	0.48 – -0.50	-0.06	0.95
Spectral Overlap	0.14	0.22	0.57 – -0.29	0.65	0.52
Vegetation	0.08	0.16	0.39 – -0.23	0.54	0.59
Elevation	-0.81	0.23	-0.36 – -1.26	-3.61	<0.01
Ordinal date	-0.06	0.10	0.14 – -0.26	-0.57	0.57
Ordinal date^2	0.30	0.12	0.54 – 0.06	2.55	0.01
Year	-0.11	0.27	0.42 – -0.64	-0.39	0.69
SPL:Background Freq	0.20	0.30	0.79 – -0.39	0.66	0.51
SPL:Spectral Overlap	-0.14	0.26	0.37 – -0.65	-0.55	0.58

Common	Latin
Orange-crowned Warbler	*Vermivora celata*

Predictors	Estimates	SE	*CI*	Z	*p*
Intercept	-4.38	0.35	-3.69 – -5.07	-12.54	<0.01
SPL	-0.10	0.09	0.08 – -0.28	-1.13	0.26
Background Freq	0.63	0.15	0.92 – 0.34	4.33	<0.01
Spectral Overlap	0.39	0.14	0.66 – 0.12	2.79	0.01
Vegetation	0.20	0.14	0.47 – -0.07	1.42	0.16
Elevation	0.49	0.22	0.92 – 0.06	2.25	0.02
Ordinal date	-1.07	0.17	-0.74 – -1.40	-6.18	<0.01
Ordinal date^2	0.15	0.14	0.42 – -0.12	1.09	0.28
Year	0.72	0.32	1.35 – 0.09	2.26	0.02

SPL:Background Freq	0.19	0.18	0.54 – -0.16	1.07	0.29
SPL:Spectral Overlap	0.08	0.15	0.37 – -0.21	0.52	0.60

	Common		Latin		
	Red-breasted Nuthatch		*Sitta canadensis*		
Predictors	Estimates	SE	*CI*	Z	*p*
Intercept	-7.74	1.10	-5.58 – -9.90	-7.06	<0.01
SPL	-0.19	0.19	0.18 – -0.56	-0.98	0.33
Background Freq	-0.25	0.36	0.46 – -0.96	-0.70	0.49
Spectral Overlap	0.56	0.40	1.34 – -0.22	1.39	0.17
Vegetation	-0.14	0.27	0.39 – -0.67	-0.53	0.60
Elevation	0.31	0.30	0.90 – -0.28	1.04	0.30
Ordinal date	-0.55	0.19	-0.18 – -0.92	-2.86	<0.01
Ordinal date^2	0.78	0.25	1.27 – 0.29	3.17	<0.01
Year	2.22	1.08	4.34 – 0.10	2.05	0.04
SPL:Background Freq	-0.36	0.38	0.38 – -1.10	-0.96	0.34
SPL:Spectral Overlap	0.68	0.44	1.54 – -0.18	1.55	0.12

	Common		Latin		
	Ruby-crowned Kinglet		*Regulus calendula*		
Predictors	Estimates	SE	*CI*	Z	*p*
Intercept	-6.77	0.78	-5.24 – -8.30	-8.69	<0.01
SPL	0.21	0.20	0.60 – -0.18	1.06	0.29
Background Freq	-0.46	0.47	0.46 – -1.38	-0.98	0.32
Spectral Overlap	-0.41	0.32	0.22 – -1.04	-1.25	0.21
Vegetation	-0.01	0.19	0.36 – -0.38	-0.05	0.96
Elevation	0.85	0.22	1.28 – 0.42	3.88	<0.01
Ordinal date	-1.22	0.29	-0.65 – -1.79	-4.18	<0.01
Ordinal date^2	0.38	0.23	0.83 – -0.07	1.63	0.10
Year	1.41	0.79	2.96 – -0.14	1.80	0.07
SPL:Background Freq	0.50	0.29	1.07 – -0.07	1.73	0.08
SPL:Spectral Overlap	0.35	0.27	0.88 – -0.18	1.29	0.20

	Common		Latin		
	Red-naped Sapsucker		*Sphyrapicus nuchalis*		
Predictors	Estimates	SE	*CI*	Z	*p*
Intercept	-4.05	0.43	-3.21 – -4.89	-9.32	<0.01
SPL	-0.03	0.26	0.48 – -0.54	-0.12	0.91
Background Freq	-0.72	0.36	-0.01 – -1.43	-2.02	0.04

Spectral Overlap	0.73	0.38	1.47 – -0.01	1.91	0.06
Vegetation	0.23	0.27	0.76 – -0.30	0.86	0.39
Elevation	-0.18	0.31	0.43 – -0.79	-0.59	0.56
Ordinal date	0.14	0.22	0.57 – -0.29	0.63	0.53
Ordinal date^2	0.14	0.22	0.57 – -0.29	0.62	0.53
Year	-0.80	0.46	0.10 – -1.7	-1.72	0.08
SPL:Background Freq	0.03	0.54	1.09 – -1.03	0.05	0.96
SPL:Spectral Overlap	0.33	0.54	1.39 – -0.73	0.61	0.54

	Common		Latin		
	Song sparrow		*Melospiza melodia*		
Predictors	Estimates	SE	*CI*	Z	*p*
Intercept	-2.57	0.30	-1.98 – -3.16	-8.65	<0.01
SPL	-0.35	0.09	-0.17 – -0.53	-3.92	<0.01
Background Freq	-0.42	0.16	-0.11 – -0.73	-2.57	0.01
Spectral Overlap	-0.13	0.13	0.12 – -0.38	-0.97	0.33
Vegetation	0.27	0.09	0.45 – 0.09	2.89	<0.01
Elevation	-0.49	0.23	-0.04 – -0.94	-2.11	0.04
Ordinal date	0.07	0.06	0.19 – -0.05	1.16	0.25
Ordinal date^2	0.11	0.07	0.25 – -0.03	1.59	0.11
Year	-0.16	0.14	0.11 – -0.43	-1.15	0.25
SPL:Background Freq	-0.23	0.16	0.08 – -0.54	-1.46	0.15
SPL:Spectral Overlap	-0.08	0.13	0.17 – -0.33	-0.63	0.53

	Common		Latin		
	Spotted Towhee		*Pipilo maculatus*		
Predictors	Estimates	SE	*CI*	Z	*p*
Intercept	-4.35	0.40	-3.57 – -5.13	-10.73	<0.01
SPL	-0.13	0.11	0.09 – -0.35	-1.14	0.25
Background Freq	-0.27	0.12	-0.03 – -0.51	-2.26	0.02
Spectral Overlap	0.19	0.09	0.37 – 0.01	2.05	0.04
Vegetation	0.19	0.17	0.52 – -0.14	1.17	0.24
Elevation	-1.18	0.29	-0.61 – -1.75	-4.03	<0.01
Ordinal date	0.01	0.08	0.17 – -0.15	0.11	0.91
Ordinal date^2	0.14	0.11	0.36 – -0.08	1.33	0.18
Year	0.76	0.24	1.23 – 0.29	3.12	<0.01
SPL:Background Freq	-0.07	0.11	0.15 – -0.29	-0.63	0.53
SPL:Spectral Overlap	-0.13	0.09	0.05 – -0.31	-1.47	0.14

	Common		Latin		
	Warbling vireo		*Vireo gilvus*		
Predictors	Estimates	SE	*CI*	Z	*p*
Intercept	-1.84	0.22	-1.41 – -2.27	-8.22	<0.01
SPL	0.03	0.06	0.15 – -0.09	0.42	0.68
Background Freq	-0.25	0.09	-0.07 – -0.43	-2.89	<0.01
Spectral Overlap	-0.02	0.07	0.12 – -0.16	-0.33	0.74
Vegetation	0.06	0.09	0.24 – -0.12	0.71	0.48
Elevation	0.02	0.20	0.41 – -0.37	0.08	0.94
Ordinal date	0.68	0.08	0.84 – 0.52	8.51	<0.01
Ordinal date^2	-0.85	0.09	-0.67 – -1.03	-9.69	<0.01
Year	0.57	0.13	0.82 – 0.32	4.48	<0.01
SPL:Background Freq	0.03	0.07	0.17 – -0.11	0.48	0.63
SPL:Spectral Overlap	0.11	0.07	0.25 – -0.03	1.50	0.13

	Common		Latin		
	Western Meadowlark		*Sturnella neglecta*		
Predictors	Estimates	SE	*CI*	Z	*p*
Intercept	-6.02	0.50	-5.04 – -7.00	-12.05	<0.01
SPL	0.13	0.17	0.46 – -0.20	0.73	0.46
Background Freq	-0.01	0.25	0.48 – -0.50	-0.04	0.97
Spectral Overlap	-0.02	0.24	0.45 – -0.49	-0.10	0.92
Vegetation	-0.31	0.17	0.02 – -0.64	-1.78	0.07
Elevation	-1.76	0.27	-1.23 – -2.29	-6.54	<0.01
Ordinal date	-0.53	0.16	-0.22 – -0.84	-3.28	<0.01
Ordinal date^2	0.55	0.18	0.90 – 0.20	3.15	<0.01
Year	0.32	0.46	1.22 – -0.58	0.69	0.49
SPL:Background Freq	0.10	0.29	0.67 – -0.47	0.33	0.74
SPL:Spectral Overlap	0.16	0.27	0.69 – -0.37	0.60	0.55

	Common		Latin		
	Western Tanager		*Piranga ludoviciana*		
Predictors	Estimates	SE	*CI*	Z	*p*
Intercept	-4.17	0.33	-3.52 – -4.82	-12.55	<0.01
SPL	-0.41	0.23	0.04 – -0.86	-1.81	0.07
Background Freq	-0.61	0.26	-0.1 – -1.12	-2.35	0.02
Spectral Overlap	-0.17	0.26	0.34 – -0.68	-0.65	0.52
Vegetation	-0.26	0.20	0.13 – -0.65	-1.30	0.19
Elevation	-0.10	0.22	0.33 – -0.53	-0.45	0.65

Ordinal date	0.56	0.18	0.91 – 0.21	3.08	<0.01
Ordinal date^2	-0.57	0.20	-0.18 – -0.96	-2.83	<0.01
Year	0.44	0.34	1.11 – -0.23	1.28	0.20
SPL:Background Freq	-0.11	0.27	0.42 – -0.64	-0.40	0.69
SPL:Spectral Overlap	-0.15	0.31	0.46 – -0.76	-0.48	0.63

	Common		Latin		
	Yellow Warbler		*Dendroica petechia*		
Predictors	Estimates	SE	*CI*	Z	*p*
Intercept	-1.56	0.26	-1.05 – -2.07	-6.08	<0.01
SPL	-0.09	0.07	0.05 – -0.23	-1.28	0.20
Background Freq	-0.17	0.12	0.07 – -0.41	-1.43	0.15
Spectral Overlap	0.02	0.11	0.24 – -0.20	0.22	0.83
Vegetation	0.11	0.09	0.29 – -0.07	1.23	0.22
Elevation	0.26	0.23	0.71 – -0.19	1.11	0.27
Ordinal date	0.04	0.07	0.18 – -0.10	0.59	0.56
Ordinal date^2	-0.61	0.08	-0.45 – -0.77	-7.89	<0.01
Year	-0.07	0.12	0.17 – -0.31	-0.56	0.58
SPL:Background Freq	-0.16	0.13	0.09 – -0.41	-1.23	0.22
SPL:Spectral Overlap	-0.14	0.12	0.10 – -0.38	-1.18	0.24

	Common		Latin		
	Yellow-rumped Warbler		*Dendroica coronata*		
Predictors	Estimates	SE	*CI*	Z	*p*
Intercept	-3.65	0.29	-3.08 – -4.22	-12.63	<0.01
SPL	0.13	0.13	0.38 – -0.12	1.03	0.30
Background Freq	0.28	0.20	0.67 – -0.11	1.43	0.15
Spectral Overlap	-0.21	0.16	0.10 – -0.52	-1.28	0.20
Vegetation	-0.17	0.14	0.10 – -0.44	-1.19	0.23
Elevation	0.76	0.19	1.13 – 0.39	3.99	<0.01
Ordinal date	-0.37	0.13	-0.12 – -0.62	-2.81	<0.01
Ordinal date^2	-0.44	0.14	-0.17 – -0.71	-3.14	<0.01
Year	1.18	0.28	1.73 – 0.63	4.18	<0.01
SPL:Background Freq	0.04	0.19	0.41 – -0.33	0.19	0.85
SPL:Spectral Overlap	0.04	0.16	0.35 – -0.27	0.24	0.81

Table S3.

Bird trait analyses summary tables. Phylogenetic generalized least squares models combined species abundance model outputs with traits to elucidate larger patterns. The top (dAIC <4) models are shown here. Bolded values indicate that variables are significant at the 95% confidence level.

Model	Intercept	Flexible	Ground	Mass	Insectivory	Peak frequency	*K*	logLik	AICc	delta	weight
SPL	-0.022	NA	NA	NA	NA	NA	2	-3.633	11.788	0.000	0.242
SPL	0.211	NA	NA	NA	NA	-0.059	3	-2.770	12.632	0.844	0.159
SPL	0.769	NA	0.003	-0.127	NA	**-0.129**	5	-0.221	13.443	1.655	0.106
SPL	-0.069	NA	0.001	NA	NA	NA	3	-3.352	13.795	2.007	0.089
SPL	-0.116	NA	NA	NA	0.024	NA	3	-3.612	14.315	2.528	0.068
SPL	0.007	NA	NA	-0.009	NA	NA	3	-3.616	14.323	2.535	0.068
SPL	-0.021	-0.002	NA	NA	NA	NA	3	-3.633	14.356	2.569	0.067
SPL	0.174	NA	0.001	NA	NA	-0.064	4	-2.315	14.535	2.747	0.061
SPL	0.448	NA	NA	-0.046	NA	-0.079	4	-2.360	14.624	2.836	0.059
SPL	0.074	NA	NA	NA	0.036	-0.060	4	-2.720	15.345	3.558	0.041
SPL	0.205	0.017	NA	NA	NA	-0.060	4	-2.758	15.420	3.633	0.039
SO	0.882	NA	NA	NA	NA	**-0.242**	3	-14.124	35.339	0.000	0.160
SO	-0.623	NA	**-0.008**	**0.273**	NA	NA	4	-12.739	35.382	0.043	0.157
SO	0.162	NA	**-0.006**	0.166	NA	-0.132	5	-11.396	35.792	0.453	0.128
SO	0.983	NA	-0.003	NA	NA	**-0.231**	4	-12.948	35.801	0.462	0.127
SO	0.219	NA	NA	NA	0.180	**-0.251**	4	-13.534	36.972	1.633	0.071
SO	-1.796	NA	**-0.007**	**0.314**	0.252	NA	5	-12.073	37.145	1.806	0.065
SO	-0.755	0.148	**-0.009**	**0.296**	NA	NA	5	-12.329	37.659	2.320	0.050
SO	0.774	NA	NA	0.020	NA	**-0.232**	4	-14.091	38.087	2.748	0.041
SO	-0.908	NA	-0.005	0.209	0.219	-0.124	6	-10.844	38.108	2.769	0.040
SO	0.888	-0.021	NA	NA	NA	**-0.240**	4	-14.116	38.136	2.797	0.040
SO	-1.128	NA	NA	0.126	0.358	**-0.194**	5	-12.693	38.387	3.048	0.035
SO	0.016	0.124	**-0.007**	0.191	NA	-0.126	6	-11.079	38.579	3.240	0.032
SO	0.826	NA	-0.003	NA	0.040	**-0.234**	5	-12.929	38.858	3.519	0.028

Model	Intercept	Flexible	Ground	Mass	Insectivory	Peak frequency	*K*	logLik	AICc	delta	weight
SO	0.978	0.025	-0.003	NA	NA	**-0.232**	5	-12.935	38.871	3.532	0.027
SPL:SO	1.693	**0.366**	0.004	**-0.397**	NA	**-0.167**	6	-0.033	16.487	0.000	0.444
SPL:SO	1.260	**0.441**	NA	**-0.299**	NA	**-0.110**	5	-2.312	17.623	1.137	0.252
SPL:SO	2.250	**0.444**	NA	**-0.355**	-0.188	**-0.127**	6	-1.381	19.182	2.696	0.115
SPL:SO	0.634	**0.413**	NA	**-0.236**	NA	NA	4	-4.763	19.431	2.944	0.102
SPL:SO	2.165	**0.377**	0.003	**-0.414**	-0.100	**-0.170**	7	0.234	19.753	3.267	0.087

Table S4.

Clay caterpillar predation model summary table. All continuous variables are centered to the mean, and standardized to one standard deviation. Predation data are analyzed with a binomial distribution (logit link).

Variable	Estimate	SE	CI	z value	p
Intercept	-2.315	0.151	-2.611 – -2.02	-15.38	<0.001
SPL	-0.441	0.101	-0.639 – -0.24	-4.375	<0.001
Background freq.	0.108	0.133	-0.153 – 0.37	0.813	0.416
Elevation	-0.027	0.178	-0.376 – 0.32	-0.150	0.880
Vegetation	-0.037	0.140	-0.311 – 0.24	-0.262	0.793
Ordinal date	0.302	0.176	-0.043 – 0.65	1.716	0.086
Bird Abundance	0.439	0.149	0.147 – 0.73	2.955	0.003
SPL:Background freq.	0.136	0.111	-0.082 – 0.35	1.233	0.217

Table S5.

Bat activity model summary tables. All continuous variables are centered to the mean, and standardized to one standard deviation. Activity count data are analyzed with a negative binomial distribution (log link).

Model: Global Bat	Frequency variable: Background frequency				
Variable	Estimate	SE	*CI*	z value	*p*
Intercept	0.063	0.569	-1.052 – 1.180	0.112	0.911
SPL	-0.082	0.018	-0.117 – -0.050	-4.631	<0.001
Background freq.	-0.206	0.020	-0.245 – -0.170	-10.291	<0.001
Year	-0.079	0.030	-0.138 – -0.020	-2.657	0.008
Elevation	0.089	0.084	-0.076 – 0.250	1.066	0.286
Vegetation	-0.229	0.030	-0.288 – -0.170	-7.768	<0.001
Moon Phase	-0.114	0.014	-0.141 – -0.090	-8.395	<0.001
Ordinal date	0.581	0.015	0.552 – 0.610	37.662	<0.001
Ordinal date^2	0.036	0.015	0.007 – 0.070	2.451	0.014
SPL:Background freq.	-0.024	0.016	-0.055 – 0.010	-1.493	0.135

Model: Global Bat	Frequency variable: Spectral difference				
Variable	Estimate	SE	*CI*	z value	*p*
Intercept	0.044	0.582	-1.097 – 1.180	0.076	0.940
SPL	-0.122	0.020	-0.161 – -0.080	-6.213	<0.001
Spectral Overlap	0.505	0.086	0.336 – 0.670	5.835	<0.001
Year	-0.006	0.028	-0.061 – 0.050	-0.202	0.840
Elevation	0.178	0.084	0.013 – 0.340	2.124	0.034
Vegetation	-0.245	0.030	-0.304 – -0.190	-8.299	<0.001
Moon Phase	-0.114	0.014	-0.141 – -0.090	-8.378	<0.001
Ordinal date	0.546	0.015	0.517 – 0.580	37.628	<0.001
Ordinal date^2	0.035	0.015	0.006 – 0.060	2.386	0.017
SPL:Spectral Overlap	-0.141	0.015	-0.170 – -0.110	-9.571	<0.001

Model: Control-only	Frequency variable: Background frequency				
Variable	Estimate	SE	*CI*	z value	*p*
Intercept	0.375	0.641	-0.881 – 1.630	0.586	0.558
SPL	0.274	0.051	0.174 – 0.370	5.410	<0.001
Background freq.	-0.215	0.038	-0.289 – -0.140	-5.651	<0.001
Year	-0.246	0.044	-0.332 – -0.160	-5.578	<0.001
Elevation	-0.235	0.170	-0.568 – 0.100	-1.379	0.168
Vegetation	-0.353	0.069	-0.488 – -0.220	-5.117	<0.001
Moon Phase	-0.040	0.021	-0.081 – 0.000	-1.921	0.055
Ordinal date	0.656	0.026	0.605 – 0.710	25.431	<0.001
Ordinal date^2	0.031	0.025	-0.018 – 0.080	1.269	0.204
SPL:Background freq.	0.171	0.033	0.106 – 0.240	5.145	<0.001

Model: Control-only	Frequency variable: Spectral difference				
Variable	Estimate	SE	*CI*	z value	*p*
Intercept	0.325	0.674	-0.996 – 1.650	0.482	0.630
SPL	0.192	0.049	0.096 – 0.290	3.920	<0.001
Spectral Overlap	1.329	0.173	0.990 – 1.670	7.671	<0.001
Year	-0.240	0.044	-0.326 – -0.150	-5.502	<0.001
Elevation	-0.513	0.149	-0.805 – -0.220	-3.454	0.001
Vegetation	-0.404	0.068	-0.537 – -0.270	-5.923	<0.001
Moon Phase	-0.049	0.021	-0.090 – -0.010	-2.410	0.016
Ordinal date	0.639	0.025	0.590 – 0.690	25.213	<0.001
Ordinal date^2	0.013	0.024	-0.034 – 0.060	0.538	0.591
SPL:Spectral Overlap	-0.190	0.022	-0.233 – -0.150	-8.669	<0.001

Model: Experimental sites only	Frequency variable: Background frequency				
Variable	Estimate	SE	*CI*	z value	*p*
Intercept	-0.325	0.588	-1.477 - 0.830	-0.553	0.580
SPL	-0.097	0.019	-0.133 - -0.060	-5.151	<0.001
Background freq.	-0.077	0.026	-0.129 - -0.030	-2.918	0.004
Year	0.255	0.043	0.170 - 0.340	5.864	<0.001
Elevation	0.123	0.094	-0.061 - 0.310	1.310	0.190
Vegetation	-0.181	0.025	-0.230 - -0.130	-7.364	<0.001
Moon Phase	-0.173	0.018	-0.208 - -0.140	-9.595	<0.001
Ordinal date	0.557	0.020	0.519 - 0.600	28.476	<0.001
Ordinal date^2	0.035	0.018	-0.001 - 0.070	1.889	0.059
SPL:Background freq.	-0.116	0.020	-0.156 - -0.080	-5.660	<0.001

Table S6.

Individual bat species model summary tables. All continuous variables are centered to the mean, and standardized to one standard deviation. Activity count data are analyzed with a negative binomial distribution (log link).

species: Antrozous pallidus

Variable	Estimate	SE	*CI*	z value	*p*
Intercept	-3.222	0.251	-3.714 – -2.73	-12.847	<0.001
SPL	-0.001	0.157	-0.309 – 0.31	-0.009	0.993
Background freq.	-0.074	0.160	-0.388 – 0.24	-0.460	0.646
Year	-0.280	0.261	-0.792 – 0.23	-1.073	0.283
Elevation	0.333	0.178	-0.016 – 0.68	1.868	0.062
Vegetation	-0.149	0.158	-0.459 – 0.16	-0.941	0.347
Moon Phase	0.042	0.115	-0.183 – 0.27	0.367	0.713
Ordinal date	0.774	0.171	0.439 – 1.11	4.536	<0.001
Ordinal date^2	-0.147	0.159	-0.459 – 0.16	-0.923	0.356
SPL:Background freq.	-0.294	0.126	-0.541 – -0.05	-2.322	0.020

species: Corynorhinus townsendii

Variable	Estimate	SE	*CI*	z value	*p*
Intercept	-3.796	0.346	-4.474 – -3.12	-10.960	<0.001
SPL	-0.446	0.192	-0.822 – -0.07	-2.323	0.020
Background freq.	-0.163	0.223	-0.60 – 0.27	-0.732	0.464
Year	0.293	0.343	-0.379 – 0.97	0.856	0.392
Elevation	0.110	0.225	-0.331 – 0.55	0.488	0.626
Vegetation	0.308	0.218	-0.119 – 0.74	1.413	0.158
Moon Phase	-0.447	0.157	-0.755 – -0.14	-2.852	0.004
Ordinal date	0.078	0.155	-0.226 – 0.38	0.502	0.616
Ordinal date^2	-0.120	0.150	-0.414 – 0.17	-0.802	0.422
SPL:Background freq.	-0.014	0.189	-0.384 – 0.36	-0.072	0.943

species: Eptesicus fuscus

Variable	Estimate	SE	*CI*	z value	*p*
Intercept	0.064	0.211	-0.35 – 0.48	0.306	0.760
SPL	-0.025	0.058	-0.139 – 0.09	-0.431	0.667
Background freq.	-0.305	0.067	-0.436 – -0.17	-4.519	<0.001
Year	-0.032	0.101	-0.23 – 0.17	-0.320	0.749
Elevation	0.640	0.177	0.293 – 0.99	3.620	<0.001
Vegetation	0.054	0.093	-0.128 – 0.24	0.580	0.562
Moon Phase	-0.068	0.047	-0.16 – 0.02	-1.453	0.146
Ordinal date	1.006	0.052	0.904 – 1.11	19.327	<0.001

Ordinal date^2	0.010	0.054	-0.096 – 0.12	0.183	0.855
SPL:Background freq.	-0.094	0.051	-0.194 – 0.01	-1.835	0.066

species: Lasiurus cinereus

Variable	Estimate	SE	*CI*	z value	*p*
Intercept	-1.333	0.278	-1.878 – -0.79	-4.792	<0.001
SPL	-0.017	0.079	-0.172 – 0.14	-0.217	0.828
Background freq.	-0.338	0.093	-0.52 – -0.16	-3.617	<0.001
Year	-0.271	0.141	-0.547 – 0.01	-1.915	0.055
Elevation	-0.099	0.220	-0.53 – 0.33	-0.450	0.653
Vegetation	-0.121	0.125	-0.366 – 0.12	-0.966	0.334
Moon Phase	0.065	0.062	-0.057 – 0.19	1.045	0.296
Ordinal date	1.032	0.077	0.881 – 1.18	13.339	<0.001
Ordinal date^2	-0.080	0.078	-0.233 – 0.07	-1.029	0.304
SPL:Background freq.	-0.131	0.069	-0.266 – 0.00	-1.891	0.059

species: Lasionycteris noctivagans

Variable	Estimate	SE	*CI*	z value	*p*
Intercept	0.331	0.379	-0.412 – 1.07	0.875	0.381
SPL	0.011	0.051	-0.089 – 0.11	0.209	0.834
Background freq.	-0.266	0.065	-0.393 – -0.14	-4.081	<0.001
Year	0.147	0.093	-0.035 – 0.33	1.575	0.115
Elevation	0.689	0.227	0.244 – 1.13	3.037	0.002
Vegetation	0.037	0.089	-0.137 – 0.21	0.411	0.681
Moon Phase	0.074	0.043	-0.01 – 0.16	1.696	0.090
Ordinal date	0.704	0.048	0.61 – 0.80	14.799	<0.001
Ordinal date^2	-0.065	0.047	-0.157 – 0.03	-1.370	0.171
SPL:Background freq.	-0.031	0.048	-0.125 – 0.06	-0.643	0.520

species: Myotis californicus

Variable	Estimate	SE	*CI*	z value	*p*
Intercept	0.520	0.199	0.13 – 0.91	2.607	0.009
SPL	-0.275	0.062	-0.397 – -0.15	-4.467	<0.001
Background freq.	-0.328	0.067	-0.459 – -0.20	-4.919	<0.001
Year	-0.113	0.099	-0.307 – 0.08	-1.139	0.255
Elevation	-0.306	0.172	-0.643 – 0.03	-1.774	0.076
Vegetation	-0.234	0.096	-0.422 – -0.05	-2.422	0.015
Moon Phase	-0.227	0.044	-0.313 – -0.14	-5.100	<0.001
Ordinal date	0.052	0.049	-0.044 – 0.15	1.045	0.296
Ordinal date^2	-0.068	0.048	-0.162 – 0.03	-1.416	0.157
SPL:Background freq.	0.055	0.052	-0.047 – 0.16	1.060	0.289

species: Myotis ciliolabrum

Variable	Estimate	SE	*CI*	z value	*p*
Intercept	1.477	0.172	1.140 – 1.810	8.611	<0.001

SPL	-0.186	0.047	-0.278 – -0.090	-3.914	<0.001
Background freq.	-0.309	0.051	-0.409 – -0.210	-6.015	<0.001
Year	-0.176	0.080	-0.333 – -0.020	-2.201	0.028
Elevation	-0.776	0.159	-1.088 – -0.460	-4.885	<0.001
Vegetation	-0.068	0.073	-0.211 – 0.080	-0.926	0.354
Moon Phase	-0.139	0.035	-0.208 – -0.070	-3.985	<0.001
Ordinal date	0.260	0.039	0.184 – 0.340	6.604	<0.001
Ordinal date^2	-0.025	0.037	-0.098 – 0.05	-0.666	0.506
SPL:Background freq.	-0.001	0.038	-0.075 – 0.07	-0.039	0.969

species: Myotis evotis

Variable	Estimate	SE	*CI*	z value	*p*
Intercept	1.695	0.201	1.301 – 2.09	8.431	<0.001
SPL	-0.002	0.042	-0.084 – 0.08	-0.043	0.965
Background freq.	0.008	0.046	-0.082 – 0.1	0.176	0.860
Year	-0.058	0.068	-0.191 – 0.08	-0.849	0.396
Elevation	-0.202	0.142	-0.48 – 0.08	-1.419	0.156
Vegetation	-0.276	0.068	-0.409 – -0.14	-4.067	<0.001
Moon Phase	-0.240	0.031	-0.301 – -0.18	-7.819	<0.001
Ordinal date	0.606	0.034	0.539 – 0.67	17.610	<0.001
Ordinal date^2	-0.004	0.033	-0.069 – 0.06	-0.120	0.904
SPL:Background freq.	0.028	0.037	-0.045 – 0.1	0.748	0.454

species: Myotis lucifugus

Variable	Estimate	SE	*CI*	z value	*p*
Intercept	1.934	0.162	1.616 – 2.25	11.953	<0.001
SPL	-0.209	0.039	-0.285 – -0.13	-5.299	<0.001
Background freq.	-0.135	0.045	-0.223 – -0.05	-3.023	0.003
Year	-0.391	0.069	-0.526 – -0.26	-5.644	<0.001
Elevation	-0.413	0.126	-0.66 – -0.17	-3.276	0.001
Vegetation	-0.345	0.061	-0.465 – -0.23	-5.660	<0.001
Moon Phase	-0.099	0.030	-0.158 – -0.04	-3.285	0.001
Ordinal date	0.781	0.036	0.71 – 0.85	21.937	<0.001
Ordinal date^2	0.014	0.034	-0.053 – 0.08	0.409	0.683
SPL:Background freq.	-0.096	0.035	-0.165 – -0.03	-2.747	0.006

species: Myotis thysanodes

Variable	Estimate	SE	*CI*	z value	*p*
Intercept	-2.886	0.267	-3.409 – -2.36	-10.802	<0.001
SPL	0.188	0.102	-0.012 – 0.39	1.837	0.066
Background freq.	0.108	0.094	-0.076 – 0.29	1.140	0.254
Year	0.189	0.166	-0.136 – 0.51	1.139	0.255
Elevation	-0.908	0.207	-1.314 – -0.5	-4.376	<0.001
Vegetation	-0.359	0.147	-0.647 – -0.07	-2.441	0.015
Moon Phase	0.080	0.074	-0.065 – 0.23	1.075	0.282

Ordinal date	0.748	0.075	0.601 – 0.9	10.015	<0.001
Ordinal date^2	0.416	0.079	0.261 – 0.57	5.257	<0.001
SPL:Background freq.	0.066	0.072	-0.075 – 0.21	0.909	0.363

species: Myotis volans

Variable	Estimate	SE	*CI*	z value	*p*
Intercept	2.055	0.217	1.63 – 2.48	9.451	<0.001
SPL	0.008	0.041	-0.072 – 0.09	0.191	0.849
Background freq.	-0.165	0.044	-0.251 – -0.08	-3.771	<0.001
Year	-0.143	0.068	-0.276 – -0.01	-2.089	0.037
Elevation	-0.870	0.179	-1.221 – -0.52	-4.858	<0.001
Vegetation	-0.155	0.066	-0.284 – -0.03	-2.343	0.019
Moon Phase	-0.206	0.032	-0.269 – -0.14	-6.534	<0.001
Ordinal date	0.432	0.034	0.365 – 0.5	12.619	<0.001
Ordinal date^2	0.027	0.032	-0.036 – 0.09	0.825	0.409
SPL:Background freq.	-0.021	0.038	-0.095 – 0.05	-0.560	0.576

species: Myotis yumanensis

Variable	Estimate	SE	*CI*	z value	*p*
Intercept	-0.351	0.126	-0.598 – -0.1	-2.797	0.005
SPL	-0.199	0.059	-0.315 – -0.08	-3.391	0.001
Background freq.	-0.188	0.067	-0.319 – -0.06	-2.785	0.005
Year	-0.144	0.104	-0.348 – 0.06	-1.384	0.167
Elevation	-0.211	0.103	-0.413 – -0.01	-2.043	0.041
Vegetation	-0.067	0.089	-0.241 – 0.11	-0.747	0.455
Moon Phase	-0.214	0.048	-0.308 – -0.12	-4.410	<0.001
Ordinal date	0.120	0.052	0.018 – 0.22	2.307	0.021
Ordinal date^2	-0.047	0.048	-0.141 – 0.05	-0.988	0.323
SPL:Background freq.	-0.077	0.053	-0.181 – 0.03	-1.464	0.143

Table S7.

Bat foraging style switching model summary tables. All continuous variables are centered to the mean, and standardized to one standard deviation. Activity counts are analyzed with a negative binomial distribution (log link).

Model: Prey playback speaker bat visits

Variable	Estimate	SE	*CI*	z value	*p*
Intercept	-2.777	1.104	-4.941 - -0.61	-2.516	0.012
SPL	0.421	0.228	-0.026 - 0.87	1.846	0.065
Background freq.	-0.148	0.271	-0.679 - 0.38	-0.549	0.583
Spectral Overlap	-0.499	1.082	-2.620 - 1.62	-0.461	0.645
Elevation	0.098	0.242	-0.376 - 0.57	0.406	0.685
Moon Phase	-0.025	0.243	-0.501 - 0.45	-0.102	0.919
Vegetation	0.344	0.218	-0.083 - 0.77	1.576	0.115
Ordinal date	0.726	0.244	0.248 - 1.20	2.981	0.003
Ordinal date^2	0.500	0.329	-0.145 - 1.14	1.519	0.129
Predicted bats in area	0.295	0.266	-0.226 - 0.82	1.108	0.268
SPL:Background freq.	-0.376	0.143	-0.656 - -0.10	-2.637	0.008
SPL:Spectral Overlap	0.249	0.236	-0.214 - 0.71	1.052	0.293

Model: Robotic moth bat visits

Variable	Estimate	SE	*CI*	z value	*p*
Intercept	-2.096	1.499	-5.034 - 0.84	-1.398	0.162
SPL	0.492	0.254	-0.006 - 0.99	1.938	0.053
Background freq.	0.568	0.291	-0.002 - 1.14	1.949	0.051
Spectral Overlap	0.471	1.514	-2.496 - 3.44	0.311	0.756
Elevation	-0.513	0.305	-1.111 - 0.08	-1.685	0.092
Moon Phase	0.377	0.215	-0.044 - 0.80	1.753	0.080
Vegetation	-0.038	0.448	-0.916 - 0.84	-0.085	0.932
Ordinal date	1.236	0.362	0.526 - 1.95	3.413	0.001
Ordinal date^2	-0.032	0.194	-0.412 - 0.35	-0.163	0.870
Predicted bats in area	-0.184	0.337	-0.845 - 0.48	-0.546	0.585
SPL:Background freq.	0.747	0.310	0.139 - 1.35	2.411	0.016
SPL:Spectral Overlap	0.560	0.422	-0.267 - 1.39	1.328	0.184

Table S8.

Bat trait analyses summary tables. Phylogenetic generalized least squares models combined species activity model outputs with traits to elucidate larger patterns. The top (dAIC <4) models are shown here. Bolded values indicate that variables are significant at the 95% confidence level. Flexible indicates that bats are able to forage via both active echolocation and passive listening.

Model	Intercept	Flexible	Mass	Peak Frequency	*K*	logLik	AICc	delta	weight
SPL	0.638	NA	NA	**-0.019**	3	9.951	-10.901	0.000	0.605
SPL	1.064	NA	-0.132	**-0.022**	4	11.880	-10.045	0.856	0.395
Freq	-0.059	**-0.216**	NA	NA	3	12.576	-16.153	0.000	0.830
Freq	0.067	**-0.224**	-0.054	NA	4	13.347	-12.979	3.174	0.170
SPL:Freq	0.284	NA	**-0.151**	NA	3	15.376	-21.752	0.000	0.849
SPL:Freq	0.459	NA	**-0.181**	-0.003	4	16.005	-18.295	3.457	0.151

Table S9.

Bird observation summary tables. Observations columns are sums of total number of times a species was observed. Unique site-days are the number of times a given species was observed as present (0 or 1) during each sampling visit (per site per day).

Common name	*Latin name*	Observations	Unique site-days
American crow	*Corvus brachyrhynchos*	88	39
American dipper	*Cinclus mexicanus*	21	6
American goldfinch	*Spinus tristis*	10	7
American kestrel	*Falco sparverius*	83	50
American robin	*Turdus migratorius*	409	176
Black-billed magpie	*Pica hudsonia*	47	17
Black-capped chickadee	*Poecile atricapillus*	75	27
Brown-headed cowbird	*Molothrus ater*	80	36
Black-headed grosbeak	*Pheucticus melanocephalus*	21	19
Brewer's blackbird	*Euphagus cyanocephalus*	52	17
Brown creeper	*Certhia americana*	5	2
Brewer's sparrow	*Spizella breweri*	52	29
Bullock's oriole	*Icterus bullockii*	109	54
Cassin's finch	*Haemorhous cassinii*	1	1
Canada goose	*Branta canadensis*	1	1
Cassin's vireo	*Vireo cassinii*	4	4
Cedar waxwing	*Bombycilla cedrorum*	22	5
Chipping sparrow	*Spizella passerina*	60	26
Cordilleran flycatcher	*Empidonax occidentalis*	9	4
Common merganser	*Mergus merganser*	2	2
Dark-eyed junco	*Junco hyemalis*	63	40
Downy woodpecker	*Dryobates pubescens*	6	4
Dusky flycatcher	*Empidonax oberholseri*	126	83
Dusky grouse	*Dendragapus obscurus*	2	2
Eastern kingbird	*Tyrannus tyrannus*	12	3
European starling	*Sturnus vulgaris*	129	26
Fox sparrow	*Passerella iliaca*	10	9
Golden-crowned kinglet	*Regulus satrapa*	4	2
Golden eagle	*Aquila chrysaetos*	1	1
Gray catbird	*Dumetella carolinensis*	2	1
Green-tailed towhee	*Pipilo chlorurus*	16	12
Hammond's flycatcher	*Empidonax hammondii*	31	17
Hairy woodpecker	*Dryobates villosus*	9	9
Hermit thrush	*Catharus guttatus*	13	6

Common name	*Latin name*	Observations	Unique site-days
House wren	*Troglodytes aedon*	864	280
Lazuli bunting	*Passerina amoena*	758	266
Lesser goldfinch	*Spinus psaltria*	1	1
Lewis's woodpecker	*Melanerpes lewis*	139	57
Lincoln's sparrow	*Melospiza lincolnii*	3	3
Mallard	*Anas platyrhynchos*	7	3
MacGillivray's warbler	*Geothlypis tolmiei*	293	137
Mountain bluebird	*Sialia currucoides*	4	1
Mountain chickadee	*Poecile gambeli*	55	22
Mourning dove	*Zenaida macroura*	60	29
Northern flicker	*Colaptes auratus*	287	127
Northern harrier	*Circus hudsonius*	4	4
Orange-crowned warbler	*Vermivora celata*	192	102
Pine siskin	*Spinus pinus*	12	5
Pilleated woodpecker	*Dryocopus pileatus*	1	1
Red-breasted nuthatch	*Sitta canadensis*	57	33
Ruby-crowned kinglet	*Regulus calendula*	89	53
Red-naped sapsucker	*Sphyrapicus nuchalis*	77	39
Rock wren	*Salpinctes obsoletus*	2	1
Red-tailed hawk	*Buteo jamaicensis*	3	2
Red-winged blackbird	*Agelaius phoeniceus*	136	21
song sparrow	*Melospiza melodia*	461	207
Spotted sandpiper	*Actitis macularius*	9	4
Spotted towhee	*Pipilo maculatus*	238	116
Steller's jay	*Cyanocitta stelleri*	1	1
Swainson's thrush	*Catharus ustulatus*	11	7
Tree swallow	*Tachycineta bicolor*	40	20
Vesper sparrow	*Pooecetes gramineus*	5	4
Violet-green swallow	*Tachycineta thalassina*	67	16
Warbling vireo	*Vireo gilvus*	542	225
Western kingbird	*Tyrannus verticalis*	1	1
Western meadowlark	*Sturnella neglecta*	131	61
Western tanager	*Piranga ludoviciana*	82	46
Western wood-pewee	*Contopus sordidulus*	7	5
Willow flycatcher	*Empidonax traillii*	4	3
Wilson's snipe	*Gallinago delicata*	2	1
Wilson's warbler	*Cardellina pusilla*	26	15
Yellow warbler	*Dendroica petechia*	576	246
Yellow-rumped warbler	*Dendroica coronata*	192	79
	**Total**	**7014**	**2981**

Table S10.

Bat observation summary tables. Total passes indicate the number of times a bat species was identified over the course of the study, whereas “site-night occurrences” indicates how many times a species occurred in a unique night and site.

Latin name	Total passes	Site-night occurrences
Antrozous pallidus	116	94
Corynorhinus townsendii	91	65
Eptesicus fuscus	6111	923
Lasiurus cinereus	1822	474
Lasionycteris noctivagans	10520	1085
Myotis californicus	4871	989
Myotis ciliolabrum	16215	1440
Myotis evotis	18025	1619
Myotis lucifugus	21889	1636
Myotis thysanodes	554	279
Myotis volans	21439	1682
Myotis yumanensis	1587	694
**Total**	**103240**	**10980**

Table S11

AIC table for bird removal model. Model 19, the scaled, quadratic version of time after sunrise and the background sound pressure level during counts (LEQ) was most predictive of when birds were more likely to be singing (dAIC =0). Additionally, Model 5, is the best fit model that doesn't contain LEQ, which was paired with our point count detectability experiment as an alternative method of controlling for background noise levels during counts.

Mod	Predictors					*K*	AIC	dAIC
0	1	-	-	-	-	1	854.55	4.39
1	ordinal date	-	-	-	-	2	856.41	6.25
2	-	TASR	-	-	-	2	856.52	6.35
3	ordinal date	TASR	-	-	-	3	858.37	8.21
4	-	-	(ordinal date)^2	-	-	2	856.35	6.19
**5**	**-**	**-**	**-**	**(TASR)^2**	**-**	**2**	**851.27**	**1.11**
6	-	-	(ordinal date)^2	(TASR)^2	-	3	853.12	2.95
7	ordinal date	-	(ordinal date)^2	-	-	3	856.94	6.78
8	ordinal date	-	-	(TASR)^2	-	3	853.11	2.95
9	-	TASR	(ordinal date)^2	-	-	3	858.32	8.16
10	-	TASR	-	(TASR)^2	-	3	852.92	2.75
11	ordinal date	TASR	-	(TASR)^2	-	4	854.75	4.58
12	ordinal date	TASR	(ordinal date)^2	-	-	4	858.93	8.77
13	ordinal date	TASR	(ordinal date)^2	(TASR)^2	-	5	855.2	5.03
14	-	-	-	-	LEQ	2	853.92	3.76
15	ordinal date	-	-	-	LEQ	3	855.53	5.37
16	-	TASR	-	-	LEQ	3	855.82	5.65
17	ordinal date	TASR	-	-	LEQ	4	857.41	7.25
18	-	-	(ordinal date)^2	-	LEQ	3	855.92	5.76

Mod	Predictors					*K*	AIC	dAIC
**19**	**-**	**-**	**-**	**$(TASR)^2$**	**LEQ**	**3**	**850.16**	**0**
20	-	-	$(ordinal\ date)^2$	$(TASR)^2$	LEQ	4	852.16	1.99
21	ordinal date	-	$(ordinal\ date)^2$	-	LEQ	4	856.83	6.66
22	ordinal date	-	-	$(TASR)^2$	LEQ	4	851.72	1.55
23	-	TASR	$(ordinal\ date)^2$	-	LEQ	4	857.81	7.65
24	-	TASR	-	$(TASR)^2$	LEQ	4	851.94	1.78
25	ordinal date	TASR	-	$(TASR)^2$	LEQ	5	853.48	3.32
26	ordinal date	TASR	$(ordinal\ date)^2$	-	LEQ	5	858.77	8.61
27	ordinal date	TASR	$(ordinal\ date)^2$	$(TASR)^2$	LEQ	6	854.86	4.7

Table S12

Number of 3-minute bird point counts by visual-only vs ‘normal’ counts.

Year	# Multimodal counts	# Vision-only counts
2017	660	670
2018	812	827
**Total**	**1472**	**1497**

Table S13

Trait table for bat data, with references as superscripts on foraging style. Where (1) gleaning bats, (2) behaviourally flexible bats (gleaning and aerial hawking), (3) clutter-tolerant aerial hawking bats, and (4) open space aerial hawking bats.

Species	Foraging style	Recorded calls	Peak frequency Mean ± SD (kHz)	Calls per hour Mean ± SD
Antrozous pallidus	1[26]	525	33.48 ± 4.77	8.0 ± 6.0
Corynorhinus townsendii	2[26]	228	51.45 ± 18.21	6.0 ± 15.6
Eptesicus fuscus	4[26]	59,536	31.54 ± 2.54	50.1 ± 190.1
Lasiurus cinereus	4[26]	15,955	26.05 ± 3.47	59.3 ± 257.4
Lasionycteris noctivagans	3[27,28]	51,785	28.69 ± 1.75	54.8 ± 163.4
Myotis californicus	3[26]	59,539	50.80 ± 4.60	84.4 ± 350.0
Myotis ciliolabrum	3[26]	158,789	44.68 ± 1.90	109.0 ± 341.4
Myotis evotis	2[29]	111,936	39.20 ± 2.12	57.1 ± 168.6
Myotis lucifugus	2[30]	105,289	43.57 ± 2.15	54.9 ± 138.3
Myotis thysanodes	2[26]	2,039	30.38 ± 3.83	19.4 ± 19.3
Myotis volans	3[26]	165,674	45.38 ± 2.49	71.7 ± 190.0
Myotis yumanensis	3[26]	9,583	54.79 ± 6.43	21.8 ± 73.8

References

1. Dooling, R. *Avian hearing and the avoidance of wind turbines*. (2002).
2. Levenhagen, M. J. *et al.* Ecosystem services enhanced through soundscape management link people and wildlife. *People and Nature* (2020).
3. Cinto Mejia, E., McClure, C. J. & Barber, J. R. Large-scale manipulation of the acoustic environment can alter the abundance of breeding birds: Evidence from a phantom natural gas field. *Journal of Applied Ecology* **56**, 2091–2101 (2019).
4. Gomes, D. G. E. *A guide to analyzing spectral information from large sound files (with R and ffmpeg)*. (Zenodo, 2020).
5. Ligges, U. tuneR–analysis of music. (2013).
6. Sueur, J., Aubin, T. & Simonis, C. Seewave, a free modular tool for sound analysis and synthesis. *Bioacoustics* **18**, 213–226 (2008).
7. Sanders, T. A. & Edge, W. D. Breeding bird community composition in relation to riparian vegetation structure in the western United States. *The Journal of wildlife management* 461–473 (1998).
8. Grindal, S. D., Morissette, J. L. & Brigham, R. M. Concentration of bat activity in riparian habitats over an elevational gradient. *Canadian Journal of Zoology* **77**, 972–977 (1999).
9. Appel, G., López-Baucells, A., Magnusson, W. E. & Bobrowiec, P. E. D. Aerial insectivorous bat activity in relation to moonlight intensity. *Mammalian Biology* **85**, 37–46 (2017).
10. Appel, G., López-Baucells, A., Magnusson, W. E. & Bobrowiec, P. E. D. Temperature, rainfall, and moonlight intensity effects on activity of tropical insectivorous bats. *Journal of Mammalogy* **100**, 1889–1900 (2019).

11. Pacifici, K., Simons, T. R. & Pollock, K. H. Effects of vegetation and background noise on the detection process in auditory avian point-count surveys. *The Auk* **125**, 600–607 (2008).

12. Hutto, R. L. Should scientists be required to use a model-based solution to adjust for possible distance-based detectability bias? *Ecological Applications* **26**, 1287–1294 (2016).

13. Ortega, C. P. & Francis, C. D. Chapter 7: Effects of gas-well-compressor noise on the ability to detect birds during surveys in northwest New Mexico. *Ornithological Monographs* **74**, 78–90 (2012).

14. Sólymos, P. *et al.* Calibrating indices of avian density from non-standardized survey data: making the most of a messy situation. *Methods in Ecology and Evolution* **4**, 1047–1058 (2013).

15. Sólymos, P. *et al.* Evaluating time-removal models for estimating availability of boreal birds during point count surveys: Sample size requirements and model complexity. *The Condor: Ornithological Applications* **120**, 765–786 (2018).

16. Sólymos, P., Moreno, M. & Lele, S. R. *detect: Analyzing wildlife data with detection error. R package version 0.3-2*. (2014).

17. Francis, C. D., Ortega, C. P., Kennedy, R. I. & Nylander, P. J. Chapter 9: Are nest predators absent from noisy areas or unable to locate nests? *Ornithological Monographs* **74**, 101–110 (2012).

18. Zhao, L. *et al.* Sometimes noise is beneficial: stream noise informs vocal communication in the little torrent frog Amolops torrentis. *J Ethol* **35**, 259–267 (2017).

19. Klump, G. M. & Larsen, O. N. Azimuthal sound localization in the European starling (Sturnus vulgaris). *Journal of Comparative Physiology A* **170**, 243–251 (1992).

20. Dominoni, D. M. *et al.* Why conservation biology can benefit from sensory ecology. *Nature Ecology & Evolution* 1–10 (2020).

21. Tuttle, M. D. & Ryan, M. J. The role of synchronized calling, ambient light, and ambient noise, in anti-bat-predator behavior of a treefrog. *Behavioral Ecology and Sociobiology* **11**, 125–131 (1982).

22. Roslin, T. *et al.* Higher predation risk for insect prey at low latitudes and elevations. *Science* **356**, 742–744 (2017).

23. Froidevaux, J. S., Fialas, P. C. & Jones, G. Catching insects while recording bats: impacts of light trapping on acoustic sampling. *Remote Sensing in Ecology and Conservation* **4**, 240–247 (2018).

24. Surlykke, A. & Kalko, E. K. Echolocating bats cry out loud to detect their prey. *PLoS one* **3**, (2008).

25. Gomes, D. G. E., Francis, C. D. & Barber, J. R. Using Past to Understand Present: Coping with Natural and Anthropogenic Noise. *BioScience* **biaa161**, 1–12 (2021).

26. Gordon, R. *et al.* Molecular diet analysis finds an insectivorous desert bat community dominated by resource sharing despite diverse echolocation and foraging strategies. *Ecology and Evolution* **9**, 3117–3129 (2019).

27. Kunz, T. H. Lasionycteris noctivagans. *Mammalian Species* 1–5 (1982).

28. Barclay, R. M. Long-versus short-range foraging strategies of hoary (Lasiurus cinereus) and silver-haired (Lasionycteris noctivagans) bats and the consequences for prey selection. *Canadian Journal of Zoology* **63**, 2507–2515 (1985).

29. Faure, P. A. & Barclay, R. M. R. Substrate-gleaning versus aerial-hawking: plasticity in the foraging and echolocation behaviour of the long-eared bat, Myotis evotis. *Journal of Comparative Physiology A* **174**, 651–660 (1994).

30. Ratcliffe, J. M. & Dawson, J. W. Behavioural flexibility: the little brown bat, Myotis lucifugus, and the northern long-eared bat, M. septentrionalis, both glean and hawk prey. *Animal Behaviour* **66**, 847–856 (2003).
