# Supplementary material for: Phantom rivers filter birds and bats by acoustic niche
Source: Nat Commun. 2021 May 24;12:3029. doi: 10.1038/s41467-021-22390-y (PMC8144611; doi:10.1038/s41467-021-22390-y)
Supplement: Supplementary file 3 — Reporting Summary [file 41467_2021_22390_MOESM3_ESM.pdf]

nature research

Corresponding author(s): Dylan Gomes and Jesse Barber

Last updated by author(s): Feb 9, 2021

Reporting Summary

Nature Research wishes to improve the reproducibility of the work that we publish. This form provides structure for consistency and transparency in reporting. For further information on Nature Research policies, see our Editorial Policies and the Editorial Policy Checklist.

Statistics

For all statistical analyses, confirm that the following items are present in the figure legend, table legend, main text, or Methods section.

n/a	Confirmed	
☐	☒	The exact sample size (*n*) for each experimental group/condition, given as a discrete number and unit of measurement
☐	☒	A statement on whether measurements were taken from distinct samples or whether the same sample was measured repeatedly
☐	☒	The statistical test(s) used AND whether they are one- or two-sided<br>*Only common tests should be described solely by name; describe more complex techniques in the Methods section.*
☐	☒	A description of all covariates tested
☐	☒	A description of any assumptions or corrections, such as tests of normality and adjustment for multiple comparisons
☐	☒	A full description of the statistical parameters including central tendency (e.g. means) or other basic estimates (e.g. regression coefficient) AND variation (e.g. standard deviation) or associated estimates of uncertainty (e.g. confidence intervals)
☐	☒	For null hypothesis testing, the test statistic (e.g. *F*, *t*, *r*) with confidence intervals, effect sizes, degrees of freedom and *P* value noted<br>*Give P values as exact values whenever suitable.*
☒	☐	For Bayesian analysis, information on the choice of priors and Markov chain Monte Carlo settings
☐	☒	For hierarchical and complex designs, identification of the appropriate level for tests and full reporting of outcomes
☒	☐	Estimates of effect sizes (e.g. Cohen's *d*, Pearson's *r*), indicating how they were calculated

Our web collection on statistics for biologists contains articles on many of the points above.

Software and code

Policy information about availability of computer code

Data collection	We used custom software in the programming language R and the package ‘FFmpeg’ in command prompt to convert 106,769 hours of long-term recordings into 71,282 individual 3-minute files starting each hour of the day (Fig S5). Thus 24, 3-minute files were created per acoustic recording location per day (one for every hour). We then used the packages `tuneR` and `seewave` to read in and measure the median frequency of sound files, respectively (see supplement for references). These hourly metrics were then averaged by date to create a daily metric. We converted 106,769 hours of long-term ARU recordings into daily-averaged median sound pressure levels (L50; measured as dBA rel. 20 µPa) using custom software ‘AUDIO2NVSPL’ and ‘Acoustic Monitoring Toolbox’ (Damon Joyce, Natural Sounds and Night Skies Division, National Park Service).
Data analysis	All models of abundance, activity, and foraging transects were generalized linear mixed effects models (glmm) in R using the package `lme4` or `glmmTMB`. All distribution families were selected based on theoretical sampling processes of the data, models were checked for collinearity, and model fits were visually checked with residual plots (see supplemental R code).

For manuscripts utilizing custom algorithms or software that are central to the research but not yet described in published literature, software must be made available to editors and reviewers. We strongly encourage code deposition in a community repository (e.g. GitHub). See the Nature Research guidelines for submitting code & software for further information.

Data

Policy information about availability of data

All manuscripts must include a data availability statement. This statement should provide the following information, where applicable:

- Accession codes, unique identifiers, or web links for publicly available datasets
- A list of figures that have associated raw data
- A description of any restrictions on data availability

All data, code, and materials used in the analysis are available as a fully-reproducible workflow from Dryad Data Repository (https://doi.org/10.5061/dryad.n5tb2rbsv).

Field-specific reporting

Please select the one below that is the best fit for your research. If you are not sure, read the appropriate sections before making your selection.

☐ Life sciences ☐ Behavioural & social sciences ☒ Ecological, evolutionary & environmental sciences

For a reference copy of the document with all sections, see nature.com/documents/nr-reporting-summary-flat.pdf

Ecological, evolutionary & environmental sciences study design

All studies must disclose on these points even when the disclosure is negative.

Study description	Twenty sites, across five drainages, within the Pioneer Mountains of Idaho were selected and matched for elevation and riparian habitat. These 20 sites were split into 10 noise playback sites, and 10 control sites (Fig S1 in supplement). The control sites ranged from quiet, slow-moving streams to relatively loud whitewater torrents. Noise playback sites, on the other hand, were relatively quiet (not whitewater) sites, where we broadcast loud whitewater river recordings with speaker arrays. At five of the noise playback sites we broadcast normal river noise (hereafter referred to as 'river noise' sites), and at the other five noise sites we broadcast spectrally altered river recordings (hereafter referred to as 'shifted noise' sites). Each site contained three locations (20 sites x 3 locations each = 60 locations total) where we continuously monitored bird abundance and bat activity for 3 months (May-July) in each of two years (2017 & 2018). This amounted to approximately 150 point count hours and 500,000 bat call sequences.
Research sample	We conducted 1330 point-counts from 28 May to 20 July 2017 and 1639 point-count events occurred from 7 May to 24 July in 2018. We deployed a total of 720 clay caterpillars throughout the 2018 breeding season.<br>We measured bat activity using Song Meter 3 (hereafter "SM3") recording units (Wildlife Acoustics Inc., Massachusetts, USA) equipped with a single SMU (Wildlife Acoustics Inc.) ultrasonic microphone. One recording unit was used at each site and pseudo-randomly rotated between the three point-count locations so that each location was monitored for at least 21 days. SM3 bat recorders obtained over 500,000 call sequences from the 20 recorders we deployed across 60 locations.
Sampling strategy	Each location (n = 60) was sampled by point counters throughout both field seasons. For bat activity data, we rotated SM3 recorders (See below) to each location (monitoring each location for at least 21 days throughout each season).
Data collection	We conducted three-minute avian point counts between one half hour before sunrise and 6 hours after sunrise (roughly 0530 – 1130 hours). During the project, we conducted 1330 point-counts from 28 May to 20 July 2017 and 1639 point-count events occurred from 7 May to 24 July in 2018.<br><br>We deployed a total of 720 clay caterpillars throughout the 2018 breeding season, following the methods of Roslin et al. (2017) to create and score predator marks on the caterpillars. Forty caterpillars were glued to stems and branches of trees between 1 - 2.5 m high at each site. Twenty caterpillars surrounded the middle point count location at each site (a set of 10 were placed upstream, and another set of 10 were placed downstream starting from the middle ARU location), while the other twenty were at upstream and downstream sampling locations (10 each at upstream and downstream locations). Each caterpillar was placed along the riparian corridor, at least 1 m apart from each other.<br><br>We measured bat activity using Song Meter 3 (hereafter "SM3") recording units (Wildlife Acoustics Inc., Massachusetts, USA) equipped with a single SMU (Wildlife Acoustics Inc.) ultrasonic microphone. One recording unit was used at each site and pseudo-randomly rotated between the three point-count locations so that each location was monitored for at least 21 days. Microphones were mounted on metal conduit at a height of approximately 3 m, and oriented perpendicular to the ground and to face away from the stream to ensure optimal recording conditions (Fig S9).<br><br>Each SM3 was programmed to automatically record bat activity for five and a half hours each night. However, the timing of these recordings differed between the 2017 and 2018 seasons. Triggered recording began 30 minutes before sunset in both years, but extended for two hours following sunset in 2017 and three hours following sunset in 2018. Triggered recording resumed three hours before dawn in both years, continuing until sunrise in 2017. However, a one-hour long recording break was programmed to occur two hours before sunrise in the 2018 season to accommodate sonic recording for a separate study (Fig S10). Ultrasonic recordings were made at a 256 kHz sample rate.<br><br>We assessed foraging-modality switching in free-flying bats using an experimental paradigm that mimicked both terrestrial and aerial prey. We hypothesized that aerial prey would be preferred under higher ambient sound levels, which was more likely to mask the cues produced by walking and calling insects (Fig S11).

Robotic insects
We used a modified version of Lazure and Fenton's (2011)27 apparatus to present bats with a fluttering target (Fig S12). This consisted of a 3 cm2 piece of masking tape affixed to a metal rod [30.48 cm length x 3.25 mm diameter], which itself was connected to a 12-volt brushed DC motor (AndyMark 9015 12V, AndyMark Inc., Kokomo, IN, USA). The no-load revolution speed of these motors (267 Hz) falls within the range of wingbeat frequency measured in Chironomidae28,29, a group that is an important food source for many North American bat species30.

We attached each motor to a tripod made of PVC piping and positioned the tripod such that the target was approximately 1.2 m above the ground. Each motor was powered by a 12 V battery (35Ah AGM; DURA12-35C, Duracell) which was controlled by a programmable 12 V timer (CN101, FAVOLCANO) to automatically start and stop the motor each night. The rotors were powered for two hours following sunset.

Prey-sound speaker playback
We created a playlist composed of several insect acoustic cues to present gleaning bats: a beetle (Tenebrio molitor) walking on dried grass, a cricket (Acheta domesticus) walking on leaves, mealworm larvae (Tenebrio molitor) on leaves, fall field cricket (Gryllus pennsylvanicus) calls, and fork-tailed bush katydid (Scudderia furcata) calls. The cricket and katydid calls were sourced from the Macaulay Library (ML527360 and ML107505, respectively).

We standardized all prey-cue recordings to an amplitude of -0.1 dB, and all to a length of four minutes, with a sample rate of 48 kHz with 16-bit depth (WAV format). To create the field playbacks, we used Adobe Audition CC 2017 to create a one-hour file which linearly combined each of the cues in random order, such that each cue was played three times per hour without occurring twice in a row. A five-second crossfade was applied between each file transition, and a five-second fade at the beginning and end of each playback. We used weatherproof speakers (Eco Extreme, Grace Digital Inc., CA, USA) and an LS-7 player (Olympus, Shinjuku, Japan) powered by a LiFePO4 battery (AA Portable Power Corp, CA, USA) housed in waterproof plastic containers to deliver the acoustic cues for passive listening bats in the field (Fig S13). Audio cues were looped continuously from dusk to dawn. Insect rustling sounds were less intense (52.1 dBF / 33.3 dBA) than cricket playback (86.4 dBF / 87.0 dBA), and the integrated hourly sound pressure level of prey playback speakers was 71.1 dBF / 71.6 dBA rel 20 µPa at 1 m in an anechoic room.

Experimental setup
Most sites received two rotors and two speakers: one of each at the center of the site, and one of each at approximately 125 m from the center of the site (in opposite directions in order to have tests in a range of acoustic environments), placed roughly 10 m from the edge of the riparian zone. Rotors and speakers at the center locations were separated by at least 50 m. The exception to this setup were the four positive control (loud whitewater river) sites, which only received a single rotor and speaker separated by 50 m because of logistical difficulties of accessing those sites. We paired each rotor and speaker with an SM2BAT+ bat detector equipped with an SMX-US microphone (Wildlife Acoustics Inc.), using tripods to elevate the microphones approximately 1 m off the ground and approximately 1 m from the speaker/rotor. We programmed the bat detectors with a gain of 36 dB and a trigger level of 18 dB to limit recordings to bats that were passing within the immediate vicinity. To allow for a comparison of activity between speakers and rotors, bat activity was only considered for the first two hours following sunset.

Timing and spatial scale

We continuously monitored bird abundance and bat activity for 3 months (May-July) in each of two years (2017 & 2018) across 60 locations. This amounted to approximately 150 point count hours and 500,000 bat call sequences.

Data exclusions

No data were excluded from analyses

Reproducibility

All attempts to repeat the experiment were successful.

Randomization

Sites were set into control, river treatment, or shifted-river treatment based on elevation and riparian-habitat matching, and due to restrictions. That is, we were unable to produce noise from some sites due to land-owner conflicts, but pseudo-randomly sorted sites based on matching elevation and vegetation.

Blinding

We checked clay caterpillars on the second, fourth, and fifth days (the first day being the placement day and the fifth day being the last day) for signs of predation. Thus, we totaled 3600 caterpillar-days during the experiment. When signs of predation were present, caterpillars were removed from the substrate and all remaining caterpillars were removed from each site and scored on the fifth day. The type of predator was assessed separately by 2-3 individuals who were blind as to which site the caterpillars came from. During all other data collection it was impossible to blind observers to the treatments, because the treatments were intense whitewater river noise that was easily heard by observers.

Did the study involve field work? ☒ Yes ☐ No

Field work, collection and transport

Field conditions

The Pioneer Mountains of Idaho are a dry sage brush desert in the foothills of the Rocky Mountains. We worked in the riparian zones of these mountains, which contained small to medium sized streams. We monitored these sites continuously from May-July in 2017 and 2018, so field conditions varied dramatically over the course of the experiment. Precipitation was generally very rare while average daytime temperatures were 18 C (min = 7 C; max = 26 C) and average nighttime temperatures were 11 C (min = 2 C; max = 25 C).

Location

Twenty sites within the Pioneer Mountains of Idaho were selected and matched for elevation and riparian habitat [spanning five drainages: Copper Creek (43.440288, -113.714253), Cottonwood Creek (43.467060, -113.635988), and Fish Creek (43.467060, -113.635988) on Lava Lake Ranch, and Trail Creek (43.755391, -114.287517) and Hyndman Creek (43.696034, -114.190139) near Sun Valley].

Access & import/export

We did not have or collect any samples that would require importing or exporting.

Disturbance	The study caused a noise disturbance across the study. This was impossible to minimize, as this was the point of the study - to measure the disturbance from this noise.

Reporting for specific materials, systems and methods

We require information from authors about some types of materials, experimental systems and methods used in many studies. Here, indicate whether each material, system or method listed is relevant to your study. If you are not sure if a list item applies to your research, read the appropriate section before selecting a response.

Materials & experimental systems

n/a	Involved in the study
☒	☐ Antibodies
☒	☐ Eukaryotic cell lines
☒	☐ Palaeontology and archaeology
☐	☒ Animals and other organisms
☒	☐ Human research participants
☒	☐ Clinical data
☒	☐ Dual use research of concern

Methods

n/a	Involved in the study
☒	☐ ChIP-seq
☒	☐ Flow cytometry
☒	☐ MRI-based neuroimaging

Animals and other organisms

Policy information about studies involving animals; ARRIVE guidelines recommended for reporting animal research

Laboratory animals	The study did not involve laboratory animals.
Wild animals	We observed birds during three-minute avian point counts. During the project, we conducted 1330 point-counts from 28 May to 20 July 2017 and 1639 point-count events occurred from 7 May to 24 July in 2018. We used Song Meter 3 (hereafter “SM3”) recording units (Wildlife Acoustics Inc., Massachusetts, USA) equipped with a single SMU (Wildlife Acoustics Inc.) ultrasonic microphone to passively record wild bats. We recorded over 500,000 bat calls.
Field-collected samples	The study did not involve field-collected samples.
Ethics oversight	All work described here was approved by the Boise State Institutional Animal Care and Use Committee (IACUC): AC15-021

Note that full information on the approval of the study protocol must also be provided in the manuscript.
